# Do Protected Areas Contribute to Health and Well-Being? A Cross-Cultural Comparison

**DOI:** 10.3390/ijerph16071172

**Published:** 2019-04-01

**Authors:** Alexandra Jiricka-Pürrer, Valeria Tadini, Boris Salak, Karolina Taczanowska, Andrzej Tucki, Giulio Senes

**Affiliations:** 1 Institute of Landscape Development, Recreation and Conservation Planning, BOKU Vienna, Peter-Jordan-Straße 65, 1180 Vienna, Austria; karolina.taczanowska@boku.ac.at; 2 Department of Agricultural and Environmental Sciences, University of Milan, Via Celoria 2, 20133 Milano, Italy; vale1990@gmail.com (V.T.); giulio.senes@unimi.it (G.S.); 3 WSL—Eidg. Forschungsanstalt, Zürcherstrasse 111, 8903 Birmensdorf, Switzerland; boris.salak@wsl.ch; 4 Maria Curie-Sklodowska University in Lublin, Plac Marii Curie-Skłodowskiej 5, 20-031 Lublin, Poland; andrzej.tucki@poczta.umcs.lublin.pl

**Keywords:** protected areas (PAs), health and well-being, ecosystem services, cross-cultural, emotional health, physical health

## Abstract

Due to their valuable landscape and natural characteristics, protected areas (PAs) distinguish themselves from other green spaces. Studies that survey individuals on the perceived capacity of PAs to provide health benefits are very limited. However, the importance of PAs for societal health could emphasize the necessity to preserve them. In addition, studies of cross-country comparisons of nature-based activities show strong cultural differences with regard to the importance of wild nature and landscape preferences. Cross-country comparisons of the perception of PAs as a resource for personal well-being and health are lacking. An extensive survey with face-to-face questionnaires was conducted in PAs in Poland, Austria, and Italy with an overall sample size of 1390 people. It examined both emotional and physical personal well-being in the context of a stay in a PA. In general, the results showed that between the three countries, different perceptions of the capacity of PAs to contribute to personal health and well-being exist. Both diverse emotional and physical benefits were associated with a stay in a PA. Moreover, respondents from the three countries assessed the perceived capacity of a PA to reduce negative physical symptoms, such as muscular pain or tension, and negative emotional symptoms, such as depression or stress.

## 1. Introduction

The health and well-being effects of contact with nature and different natural settings have been the subject of several studies over the past thirty years (e.g., [1,2,3,4,5,6]). For recreational purposes, natural ecosystems play an important role as places where people can go to refresh themselves [7]. Staats et al. [1] refer to the change in mood in connection to specific forest landscape characteristics. They built on previous studies on the influence of landscape structures (density, accessibility, etc.) on an emotional level [8,9,10,11]. The Japanese research on Shinrin-Yoku has contributed to understanding the particular effect of forest landscapes on diverse health determinants [12,13,14].

On the mental (emotional) dimension, studies have dealt with exposure to nature and nature-bound activities in different types of landscapes and with regard to the regulation of stress, the ability to concentrate, short-term memory, and irritability [15,16,17,18,19], as well as fatigue and the recuperation of mental health [4,20]. A large meta-analysis on medical parameters of physical health was addressed by Twohig-Bennett and Jones [21].

While many studies have examined green structures in urban environments and their impact on physical well-being and activity in peoples’ daily lives [22,23,24,25,26], others have revealed landscape preferences related to activities outside city areas [27,28]. Studies such as those by Özgüner and Kendle [29] and Payne et al. [30] deal with preferences of designed and easily accessible landscapes/parks versus more wild and near-nature settings. Among others, Humpel et al. [24], Leslie et al. [31], Saelens et al. [32], and Titze et al. [33] have studied the influence of aesthetic values and qualities of landscapes.

Protected areas (PAs) are wild, semi-wild, or cultural landscapes with a high recreational value. Due to their valuable landscape and natural characteristics, PAs distinguish themselves from other green spaces. Whereas profound knowledge has been gained about landscape preferences in PAs (e.g., [34]), studies that survey individuals on the perceived capacity for PAs to provide health benefits are very limited [35,36,37,38]. Watson et al. [38] mention the specific value of PAs as resources for public health and describe management actions to increase this potential. Puhakka et al. [39] confirmed the perceived impact on mental and physical health for visits in Finnish PAs. Terraube et al. [40] point out the multiple effects of PAs in the delivery of ecosystem services and in particular their role in health and wellbeing.

The perception of green areas varies, however, not only because of their landscape characteristics but also because of impact factors related to the visitor’s personal background. In this regard, the impact of demographic characteristics such as gender, age, and ethnic affiliation/background on access to green structures and preferences in utilization behavior has been examined in studies by Kaspar and Bühler [41], Maas et al. [42], Lee et al. [25], Payne et al. [30], and Wilbur et al. [43], among others. In addition, studies about cross-country comparisons of nature-based activities such as those by Landauer et al. [44,45] showed strong cultural differences with regard to the importance of wild nature and landscape preferences. Wynveen et al. [46] examined cross-cultural differences of place attachment. So far, however, the perception of PAs as a resource for personal well-being and health has scarcely been examined via cross-country comparison. Thus, this paper, evolving from an international COST-network on the impact of ecosystem services on health and well-being, will compare three cultural backgrounds (Mediterranean, Central European, and Eastern European) represented by three countries (Austria, Italy, and Poland) in order to identify possible differences in the attitude toward PAs and their capacity to contribute to health and well-being.

Three main underlying hypotheses will be discussed in this paper:

1. Cultural differences occur regarding motives to visit PAs.
2. The benefits associated with contact with nature vary between the three countries. Consequently, cultural differences exist regarding the perceived capacity for PAs to contribute to personal well-being, i.e., the salutogenic effect of a visit to a PA.
3. Activities carried out in good or bad health conditions differ between countries. The likelihood of visiting a PA depends on health conditions and differs between countries.

## 2. Background

Protected Areas are important in securing and delivering several aspects of the concept of Cultural Ecosystem Services (CES) described in the Millennium Assessment [47]. White et al. [48] and Kettunen et al. [49] differentiate between use and non-use values. Jackson et al. [50] contributed significantly to the field by highlighting the health benefits of diverse ecosystems through a systematic overview of scientific studies on health and ecosystem services. Recreational opportunities and tourism-related economic benefits of a natural site are the main components of use values within CES. Several studies deal with the contribution of PAs for recreational and tourism purposes [51,52,53]. Most of them including Eagles [51] or Küpfer [54] refer directly to economic impacts. Backhaus et al. [55] estimated strong direct economic impacts from National Parks in Switzerland as well as indirect effects on regional income. Job et al. [56] documented a similarly strong economic impact from the National Park Berchtesgarden in Germany. Fleischerhacker and Pauer [57] analyzed the effects of National Parks in Austria. Within their study, they confirmed a significant correlation between the presence of a national park and an increased duration of stay in the region in comparison to nearby areas outside the PA. Studies on economic impact have also been carried out for other types of PAs. Heintel and Weixelbaumer [58] and Lehar et al. [59] studied economic impacts correlated to nature parks in Austria and Italy. Both studies reveal a strong monetary impact. Brau and Cao [60], Englund [61], Kelly et al. [62], and Forster and Siegrist [63] examine the relevance of PAs with respect to destination choice. Pröbstl-Haider and Haider [64] studied the combination of PAs in the context of destination choice with the overall demand characteristics of alpine tourism. Both demand and supply sides were surveyed with regard to different types of PAs. The study built on the results of Reinius and Fredman [65] and earlier studies by Eagles [51] and Nolte [66], which compared recognitions of different types of PAs.

Individual and aesthetic values strongly influence destination choice [34,64] in the context of PAs. Despite their influence on the economic value through tourism and recreational use, aesthetics are, per se, a non-use value related to “scenic beauty” but also to attachments to places and to identity building [67]. Amongst others, Kaltenborn and Williams [68], Lin and Lockwood [69], Halpenny [70], Bonaiuto et al. [71], and Eder and Arnberger [72] examined the capacity of PAs to enhance attachments to places and identity building. Further important non-use values—also covered by PAs—can be spiritual functions, in particular with regard to sacral places and immaterial cultural heritage connected to certain natural structures [73,74].

An additional important aspect is the non-use value of individual physical, mental, and psychological wellbeing of visitors of PAs. Only a few studies have been carried out, however, referring to the effect of PA on human well-being and health [35,37,38,75]. By cross-country comparison, the current study can contribute to further insight on the impact on health and well-being perceived by visitors in PAs.

## 3. Materials and Methods

### 3.1. Study Design

An extensive survey was conducted in nine PAs in three countries (Austria, Italy, and Poland). Trained interviewees used standardized questionnaires translated into the national language (paper-and-pencil-interview (PAPI)). The PA visitors were randomly selected and interviewed on-site. For each country, different types of PAs were included in the national sub-sample data collection process in order to avoid bias by a special type of PA (National Park, Biosphere Reserve, and Nature Park, according to IUCN criteria). Data collection was carried out on two to three sampling days per study area (including at least one weekend day). Interviews took place at popular locations in the park territory (e.g., the entrance, the main hut, and the main landscape attraction point). Table 1 provides an overview of major characteristics of the studied areas in Italy, Poland, and Austria and the obtained samples.

The standardized questionnaire included 24 questions—12 of which employed Likert scales—and a commentary box at the end. Questions were partly adapted from previous research on nature, health, and well-being [71]. The design of the questionnaire used in this study can be found in the Supplementary Materials (Annex I, S1).

The first part concerned the characteristics of PA visitations in the past and on the day of data collection, including the frequency of park visits, the length of stay, motives, activities, company, knowledge of the PA category to which the park belongs, the characteristics of the park in relation to health and well-being, and the circumstances of the visit.

The second part contained questions addressing physical and mental well-being effects. Firstly, activities carried out in good and bad health conditions were examined; secondly, the effects of nature and PAs specifically were surveyed.

In the third part, demographic data regarding age, gender, education level, profession, postal code, and the distance to the park from home, were collected.

### 3.2. Description of the Sample

In total, 1392 respondents participated in the survey. The sub-samples in the specific countries had similar sizes (see Table 1 and Table 2) and an almost equal distribution among male and female visitors.

**Figure 1:**
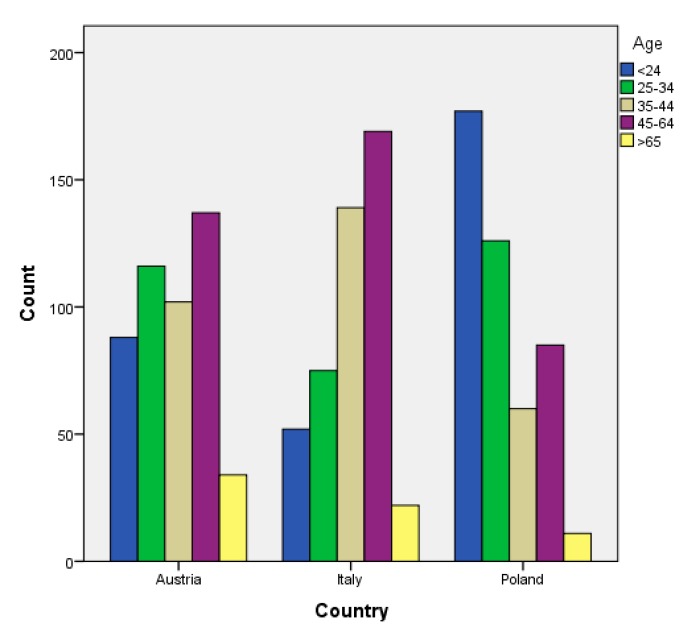
Age of the country samples.

In contrast, the age groups in the sub-samples varied significantly (see Figure 1). Differences between country samples related to an age group below 24 years, which was overrepresented in the Polish sample. According to national studies [76,77,78,79], this high amount of young people is typical, however, for the visitor structure in Poland; it mainly encompassed groups of pupils.

### 3.3. Data Analysis

For recording and evaluation of the collected data, SPSS (Pasw Statistics 18.0) was used. Mean values, frequencies, a one factorial ANOVA, and *t*-tests were the main statistic tools applied throughout the study. The zero hypotheses that were generated with the applied test were analyzed afterwards in order to adapt the outcome to the actual research questions and hypotheses of the study. Indications of the main test used for assessment are given in the figures. Significances of *p* < 0.05 or if *p* < 0.01 are indicated in the graphics or in the tables.

## 4. Results

In the following, the results are presented according to the main hypotheses presented in Section 1. Overall, several significant differences could be observed between the sub-samples, in particular with regard to the perceived capacity for PAs to contribute to human health and well-being among the countries.

### 4.1. Motives to Visit PAs

**H1:**  *Cultural differences occur regarding motives to visit Pas.*

**Figure 2:**
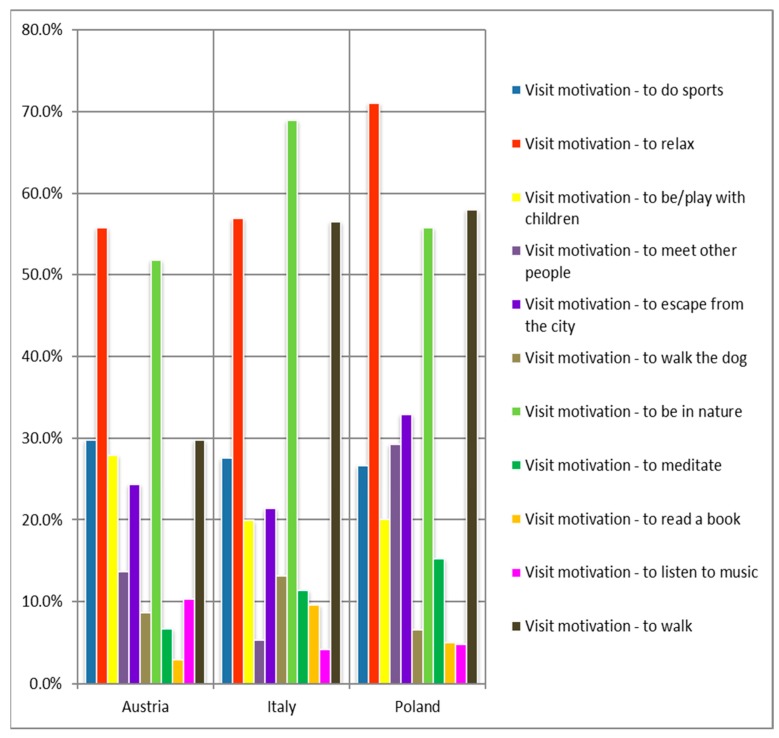
Motives to visit a protected area. Austria, *n* = 476; Italy, *n* = 457; Poland, *n* = 459 (*p* ≤ 0.01).

Comparing visitor motives (adapted from Chiesura [80]), significant differences between the three countries are apparent (see Figure 2). Whereas “relaxation” and “to be in nature” were common motives in all three countries, “walking” was a significantly less common activity in Austria than it was in the two other countries. The main motive varied as well; in Poland and Austria, the most common motive to visit a PA was “relaxation”, whereas in Italy it was “to be in nature.” For all three countries, playing sports was the 4th or 5th most common motive out of 11.

In order to investigate the health and well-being context of the motives to visit a specific PA, the circumstances for the park visits where surveyed (Annex I, survey question 6). Significant differences between the countries were apparent again (see Table 3).

**Figure 3:**
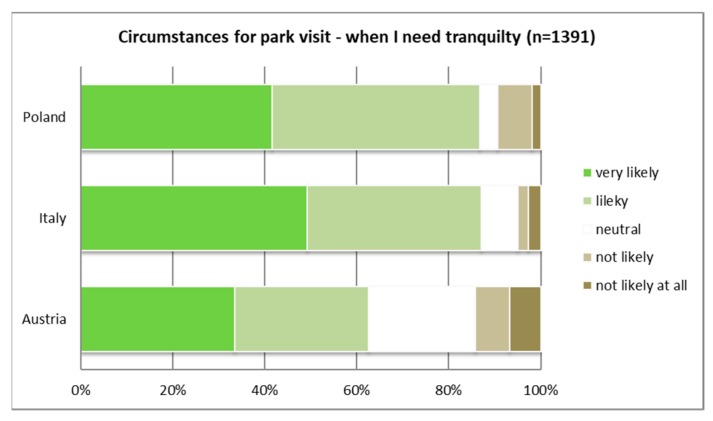
Visiting PAs to seek tranquility; cross-country comparison (*p* ≤ 0.01).

**Figure 4:**
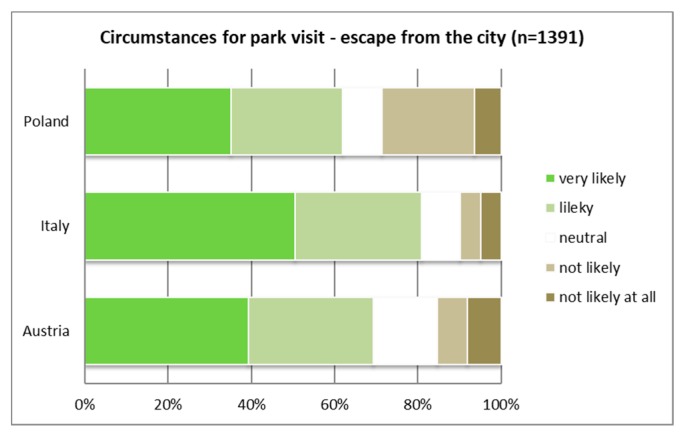
Visiting PAs to escape from the city; cross-country comparison (*p* ≤ 0.01).

The majority of Italians and Polish respondents (around 90%) would “very likely” or “likely” visit a PA when they seek tranquility (see Figure 3), whereas in Austria only two-thirds would “very likely” or “likely” visit a PA under this circumstance. Half of the Italian respondents would “very likely” visit a PA when they want to “escape from the city” (see Figure 4).

In the case of “feeling well,” more than two-thirds of both the Italian and Austrian samples said that they would “extremely likely” or “very likely” go to this PA. In contrast, only 40% of the Polish sample stated a strong likelihood. When feeling bad, mentally or emotionally, differences were not highly significant.

When looking at the age groups, significant differences when comparing the youngest visitors of the three countries exist with respect to the circumstances of visiting PAs. Italian and Polish young adults were particularly likely to visit PAs when they seek tranquility or want to escape from the city (see Supplementary Materials S2, Section 10). The “search for tranquility” was stronger among elderly visitors in general. Moreover, Italian and Polish respondents gave the highest scores with regard to the likelihood of visiting a PA under this circumstance. For the oldest age group, results were very similar in all three countries (Supplementary Materials S2, Sections 9 and 11).

### 4.2. Benefits Associated with Contact with Nature in PAs

**H2:**  *Benefits associated with contact with nature vary between the three countries. Consequently, cultural differences exist regarding the perceived capacity for PAs to contribute to personal well-being, i.e., the salutogenic effect of a visit to a PA.*

**Figure 5:**
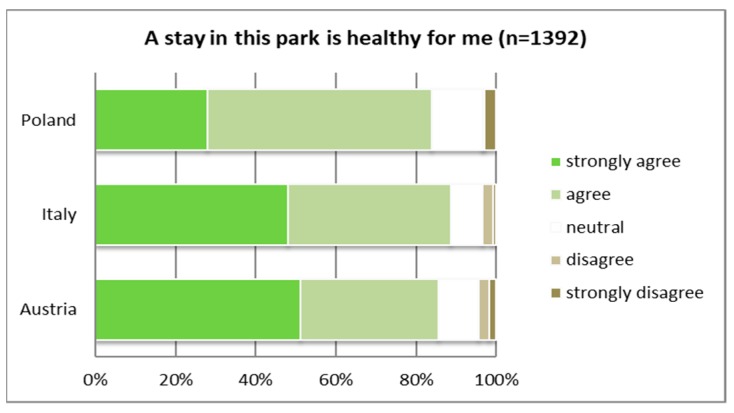
Benefits associated with a stay in the park and therefore contact with nature (*p* ≤ 0.01).

With regard to the health benefits associated with a stay in the survey area, significant differences were apparent. More than half of the Italians and Austrians strongly agreed that a stay in the specific park where the survey was conducted positively contributed to their well-being (see Figure 5), but only a little more than 20% of the Polish respondents showed the same level of agreement. Altogether, agreement with this statement (“a stay in this park is healthy for me”) reached 90% in Italy and a bit under 90% in Austria, whereas in Poland only around 60% of the respondents showed agreement at all.

**Figure 6:**
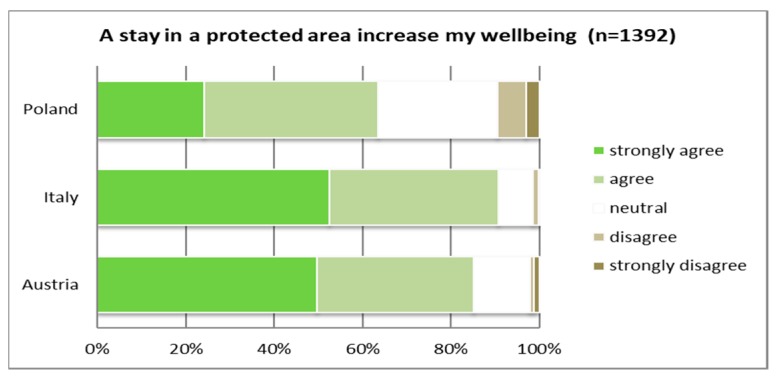
Benefits associated with a stay in a PA regarding personal well-being (*p* ≤ 0.01).

Similarly, the Polish sample considered the importance of PAs in general for personal well-being more minor than the Austrian and Italian respondents (see Figure 6). Half of the Austrian sample and even a bit more than half of the Italian participants of this survey agreed strongly to the statement “a stay in a protected area increases my well-being”.

Regarding differentiated benefits expressed in two statements, “nature makes me feel relaxed” and “to stay in contact with nature recharges my battery,” half of the Austrian and Italians in this study showed strong agreement, whereas only a third of the Polish respondents did.

### 4.3. Perceived Capacity to Contribute to Personal Well-Being through Reduction of Symptoms of Bad Health Conditions

With regard to the ability to reduce negative symptoms of both mental and physical health, significant differences between the countries were evident.

First, respondents were asked whether contact with nature in generally reduces the most prominent symptoms of “stress” and “fatigue.” Around 60% of Italian respondents agreed that contact with nature “always” reduces symptoms of stress and fatigue. In the Polish sample, it was only one-third. In all three countries, however, only around 3–5% stated that contact with nature “rarely” or “never” reduces stress and fatigue.

**Figure 7:**
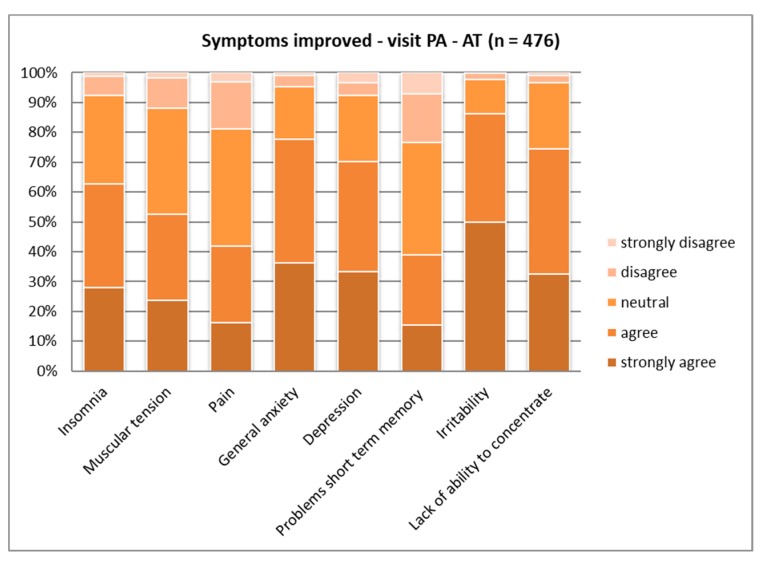
Symptoms improved by PA visitation—Austria (*p* ≤ 0.01).

**Figure 8:**
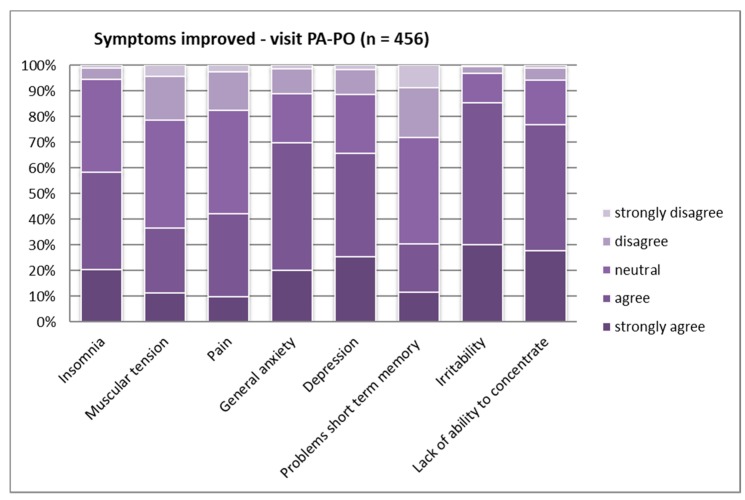
Symptoms improved by PA visitation—Poland (*p* ≤ 0.01).

**Figure 9:**
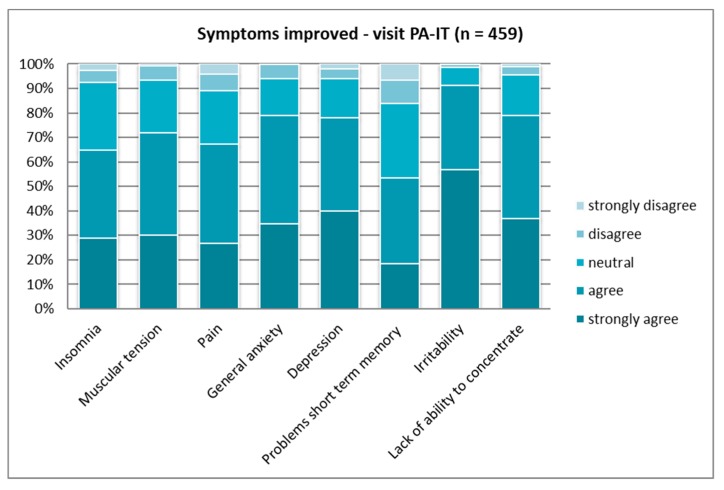
Symptoms improved by PA visitation—Italy (*p* ≤ 0.01).

When asked for more detail about personal experience with symptoms being reduced by contact with nature, significant differences became evident, except for the “lack of ability to concentrate” and “insomnia” (see Table 4). Overall, the contribution to mental health was regarded as stronger than that to physical health. All three countries experienced positive effects on the “lack of ability to concentrate” quite similarly (Figure 7, Figure 8 and Figure 9).

For all other symptoms, again the Polish respondents showed the lowest agreement in all categories (see Figure 8). Italians and Austrians believe that PAs have a high capacity (“strongly agree”) to reduce “irritability” and “depression” (around half of the samples had strong agreement). In the Polish sample, around 85% agreed that these two symptoms could be reduced (Figure 8). The Austrian and Italian samples showed similar agreement (almost 80% answered with “agree” or “strongly agree”) regarding the ability to reduce “general anxiety”; the Polish sub-sample showed 10% less agreement (see Figure 7 and Figure 9).

When looking at the age groups, highly significant differences could be found regarding the reduction of all symptoms, except “depression” (see Supplementary Materials). The data comparison between same-age groups for all three countries showed significant differences regarding the reduction of pain in the youngest groups of respondents, with the Polish sub-sample ranking the lowest.

### 4.4. Likelihood of Visiting a PA in Good or Bad Health Conditions

**H3:**  *Activities carried out in good or bad health conditions differ between countries. The likelihood of visiting a PA depends on health conditions and differs between countries.*

**Figure 10:**
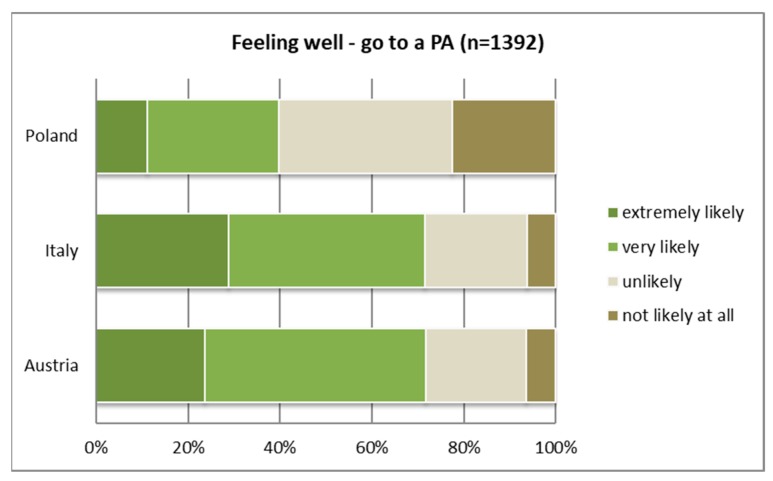
Likelihood of going to a protected area when feeling well (mentally and physically).

**Figure 11:**
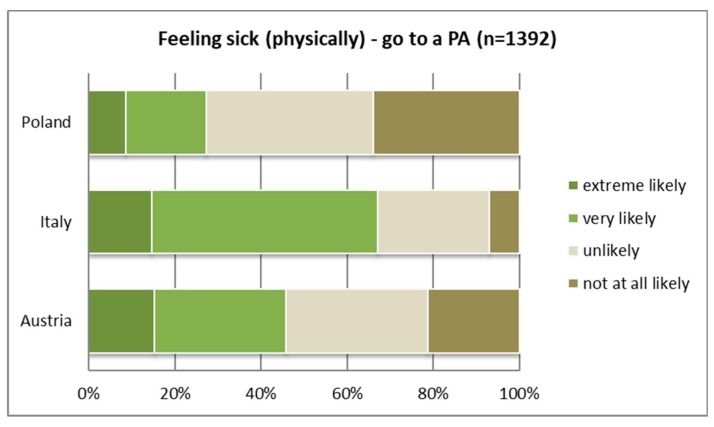
Likelihood of going to a protected area when feeling physically sick (in pain).

Activities carried out in good or bad health conditions differ significantly between the countries (see Table 5 and Table 6). Respondents in all three countries differ in the likelihood (“very” or “extremely likely”) of going to a PA and a “normal” park when “feeling well.” Interestingly, the Austrian and Italian samples responded similarly regarding the likelihood of going to a PA under good health conditions (see Figure 10) and differed only in the case of bad health conditions (see Figure 11). In the case of feeling physically sick (e.g., back pain or headache), Italians were most likely to go to a PA anyway, whereas the Austrian and Polish samples showed a different attitude. For both conditions—feeling well or feeling sick—the Polish sample showed the lowest likelihood of visiting a PA. In the case of bad health conditions, however, the likelihood of going to a park rather than a PA was higher in all three countries’ samples (see Table 6).

Table 5 shows the likelihood of visiting a PA in comparison with other activities in the case of mental and physical well-being. Under conditions of physical and mental well-being, playing sports and going to a park or PA received the highest mean values in terms of likelihood scores. In particular, the Polish sample preferred to carry out activities other than visiting a park or PA in the case of bad health conditions. The majority would either stay at home or in their living areas to carry out activities such as shopping or listening to music. On the contrary, the Italian sample showed the strongest willingness to visit PAs under conditions of illness or a lack of personal wellbeing.

A comparison between types of parks is provided in the Supplementary Materials (see Sections 2, 4, and 8 in Annex II, Supplementary Materials). Detailed analyses between the parks of the same category show results with partly significant differences. Due to the small sample size of one park per category for each country, this study could not investigate the influence of types of PAs thoroughly. The diverse characteristics of each park as well as the surrounding source area of the visitors, compared with PA category, might influence the results more strongly.

## 5. Discussion

The results of this cross-country comparison indicate significant differences, which might be caused by the cultural influence but also by the age composition of the visitors of PAs in the three countries. In general, the study results show different perceptions of the capacity of PAs to contribute to personal health and well-being between the three countries. These differences might be linked to both the diverse emotional and physical benefits associated with a stay in a PA and to differences regarding the perceived capacity of PAs to reduce negative physical symptoms such as muscular pain or tension and negative emotional symptoms such as depression or stress.

The study also shows significant differences regarding the circumstances under which a PA is visited. Whereas in the case of good health conditions and well-being, the results were quite similar, significant differences in behavior become apparent in the case of bad health conditions. The high percentage of the Italian sample that stated that they would visit PAs in the case of bad health conditions might be influenced by their strong belief in the ability of PAs to reduce symptoms of bad health. This interrelationship could be subject to further investigation.

Furthermore, differences in the demographic characteristics of PA visitors in the three countries may provide an additional reason for intercultural differences. Previous studies such as that by Payne et al. [30] showed differences in attitudes toward parks as a resource for recreation and the intention to visit them between younger and older age groups. Whereas in Italy, the average age of visitors was higher than in other countries, Polish visitors were particularly young. The high share of young people in the Polish sample might have an impact on the results, although they represent the current age composition of visitors of PAs in Poland very well [76,78]. While the comparison between age groups shows significant differences, this variable cannot entirely explain the diverse responses for all questions. Within the same age group, significant differences were found between the three countries in certain response categories.

Another important consideration is the likely influence of the type of PA on the results. Due to the small sample of one park of each category per country, the influence of the type of PA demands further attention with a larger sample for each type. The relationship between the investigated variables and the type of PA varies over the entire survey (significant differences provide no consistent pattern). In this context, the specific features and characteristics of landscape and wilderness as well as the accessibility of the PAs seem to have an influence on the perceived capacity of PAs to contribute to health and well-being. A study on the three Italian parks involved in this study and a larger Italian study parallel to this one have already discussed these influencing factors [81,82].

In contrast to the aforementioned differences regarding the perceived capacity of PAs to contribute to health and well-being, the two main motives to visit PAs are the same in all of the three countries. In all of the three countries, a stay in a PA was believed by the majority of visitors to have an overall positive impact on health and well-being. Effects on mental health, such as improving symptoms of insomnia, depression, and general anxiety were rated highly. This confirms the results of Puhakka et al. [39] regarding Finnish PAs, which highlighted the effect of visits in PAs on psychological well-being and stress reduction, and emphasizes the claims made by Terraube et al. [40] regarding the value of the role of PAs in improving health conditions of both the nearby population as well as recreation seekers and tourists.

## 6. Conclusions and Outlook

The main motives of visiting PAs were similar in the three countries, but the strongest motive (s) varied. Moreover, the perceived capacity to reduce symptoms of bad health conditions, and vice versa, contributing to improvement of health and well-being, differed significantly in this study. Regarding the impact of age on the results, this survey well reflects the age composition of visitors in each country. Nevertheless, this variable inherently influences the results. In particular, regarding symptoms of bad health conditions, the experience of those impairments in the respondents’ own life might have varied among age groups. Consequently, this might have influenced the response toward the perceived capacity to reduce those health and well-being issues.

Further research will be required to verify the cultural influence of the diverse perceptions of PAs in the context of health and well-being. In particular, the general high interest of Italians on topics of health and wellbeing as well as the tradition to be out and walking in nature could influence their expectations toward the capacities of PAs. On the contrary, the majority of Polish PAs have only become popular in the last two to three decades (except for the Tatra National Park)—a fact that could influence interest in visiting them under various circumstances, in particular in the case of an absence of health and well-being. Of major interest is the link between the perceived capacity for PAs to contribute to health and well-being and the value attributed to the protection of the areas.

## Figures and Tables

**Table 1:** List of study areas and their general characteristics.

	Study Area	Designation as PA (year)	Size (ha)	Distance to Urban Area(s), ca. (km)
Austria	Nationalpark Donau Auen	1996	9300	100
	Naturpark Oetscher Tormauren	1970	17,000	200
	Biosphere reserve Lungau	2012	15,000	300
Italy	Parco della Val Grande	1992	15,000	100
	Parco naturale dell’Alpe Veglia e dell’Alpe Devero	1995	9000	200
	Riserva della Biosfera “Valle del Ticino”	1974	92,000	50
Poland	National Park Roztocze	1974	8482	130
	Polesie National Park	1990	9762	50
	Janowskie Forests Landscape Park	1984	40,000	80

**Table 2:** Description of the country sub-samples (gender).

Total Sample		Male	Female
Austria	count	226	250
	% within country	47.4	52.4
Italy	count	228	229
	% within country	49.9	50.1
Poland	count	218	241
	% within country	46.8	52.5

**Table 3:** Cross-country comparison indicating the likelihood to visit the PA under diverse circumstances (green = highly significant differences, orange = significant differences, red = no significant differences).

Under Which Circumstances Do You Visit This Park? (Likert Scale Coding: 1—Very Likely, 2—Likely, 3—Neutral, 4—Not Likely, 5—Not Likely at All)							
			*n*	Mean	Std. Deviation	Std. Error	
Circumstances for park visit—when I need tranquility	0.000	Austria	476	2.25	1.189	0.054	
		Italy	457	1.71	0.907	0.042	
		Poland	459	1.83	0.949	0.044	
		Total	1392	1.94	1.050	0.028	
Circumstances for park visit—when I’m feeling well	0.000	Austria	476	2.07	0.965	0.044	
		Italy	457	1.86	0.863	0.040	
		Poland	459	1.92	0.830	0.039	
		Total	1392	1.95	0.893	0.024	
Circumstances for park visit—when I’m feeling bad (emotionally stressed, or depressed)	0.068	Austria	476	2.79	1.267	0.058	
		Italy	457	2.56	1.254	0.059	
		Poland	459	2.58	1.142	0.053	
		Total	1392	2.65	1.226	0.033	
Circumstances for park visit—when I want to escape from the city	0.000	Austria	476	2.15	1.245	0.057	
		Italy	457	1.83	1.098	0.051	
		Poland	459	2.38	1.328	0.062	
		Total	1392	2.12	1.247	0.033	

**Table 4:** Country comparison indicating respondents’ responses to which symptoms can be reduced by contact with nature (green = highly significant differences, orange = significant differences, red = no significant differences).

Reduction of Symptoms (ANOVA)		Sum of Squares	F	Sig.
**Irritability**	Between Groups	27.673	25.115	0.000
**Lack of ability to concentrate**	Between Groups	3462	2343	0.096
**Insomnia**	Between Groups	3416	1933	0.145
**Muscular tension**	Between Groups	120.390	63.640	0.000
**Pain**	Between Groups	85.993	43.313	0.000
**General anxiety**	Between Groups	27.731	17.508	0.000
**Depression**	Between Groups	24.006	12.431	0.000
**Short-term memory**	Between Groups	43.930	18.126	0.000

**Table 5:** Activities in the case of mental and physical well-being (green = highly significant differences, orange = significant differences, red = no significant differences).

What Do You Do When You Are Feeling Physically and Mentally Well (Happy—No Stress—Relaxed)?								
			*n*	Mean	Std. Deviation	Std. Error	95% Confidence Interval for Mean	
							Lower Bound	Upper Bound
physically and mentally well—Sleep	0.000	Austria	475	2.79	0.953	0.044	2.71	2.88
		Italy	457	2.67	0.951	0.045	2.58	2.76
		Poland	456	2.85	1.149	0.054	2.75	2.96
		Total	1388	2.77	1.023	0.027	2.72	2.83
physically and mentally well—Read a book	0.041	Austria	475	2.32	0.862	0.040	2.25	2.40
		Italy	457	2.06	0.926	0.043	1.97	2.14
		Poland	458	2.53	0.910	0.043	2.44	2.61
		Total	1390	2.30	0.919	0.025	2.26	2.35
physically and mentally well—Watch TV	0.137	Austria	474	2.60	0.879	0.040	2.52	2.68
		Italy	457	2.47	0.920	0.043	2.39	2.56
		Poland	458	2.77	0.907	0.042	2.68	2.85
		Total	1389	2.61	0.909	0.024	2.57	2.66
physically and mentally well—Sports	0.000	Austria	475	1.78	0.753	0.035	1.72	1.85
		Italy	456	1.77	0.789	0.037	1.69	1.84
		Poland	458	2.22	0.902	0.042	2.13	2.30
		Total	1389	1.92	0.842	0.023	1.88	1.97
physically and mentally well—Go to a park	0.000	Austria	475	2.07	0.876	0.040	1.99	2.15
		Italy	457	1.81	0.790	0.037	1.74	1.88
		Poland	454	2.28	0.939	0.044	2.19	2.36
		Total	1386	2.05	0.890	0.024	2.01	2.10
physically and mentally well—Go to a protected area	0.000	Austria	474	2.11	0.839	0.039	2.04	2.19
		Italy	457	2.06	0.873	0.041	1.98	2.14
		Poland	457	2.72	0.943	0.044	2.64	2.81
		Total	1388	2.30	0.934	0.025	2.25	2.34
physically and mentally well—Take a walk in the city	0.000	Austria	474	2.53	0.929	0.043	2.45	2.61
		Italy	457	2.16	0.881	0.041	2.07	2.24
		Poland	452	2.54	1.025	0.048	2.45	2.64
		Total	1383	2.41	0.963	0.026	2.36	2.46
physically and mentally well—Go shopping	0.009	Austria	475	2.46	0.946	0.043	2.37	2.54
		Italy	457	2.26	0.951	0.044	2.17	2.35
		Poland	456	2.46	1.030	0.048	2.36	2.55
		Total	1388	2.39	0.980	0.026	2.34	2.44
physically and mentally well—Listen to music	0.000	Austria	475	1.89	0.845	0.039	1.82	1.97
		Italy	457	1.84	0.769	0.036	1.77	1.91
		Poland	458	2.18	0.980	0.046	2.09	2.27
		Total	1390	1.97	0.881	0.024	1.92	2.02

**Table 6:** Activities in the case of physical/mental absence of well-being and health (green = highly significant differences, orange = significant differences, red = no significant differences).

What Do You Do When You Are Feeling Physically Sick (Headache-Backache)/Mentally Sick (Stress—Fatigue)?								
			*n*	Mean	Std. Deviation	Std. Error	95% Confidence Interval for Mean	
							Lower Bound	Upper Bound
physically sick—Sleep	0.000	Austria	475	1.75	0.800	0.037	1.68	1.82
		Italy	457	1.72	0.824	0.039	1.65	1.80
		Poland	459	1.87	1.024	0.048	1.78	1.97
		Total	1391	1.78	0.889	0.024	1.74	1.83
mentally sick—Sleep	0.000	Austria	475	1.92	0.855	0.039	1.85	2.00
		Italy	457	1.86	0.879	0.041	1.77	1.94
		Poland	459	0.00	0.000	0.000	0.00	0.00
		Total	1391	1.27	1.137	0.030	1.21	1.33
physically sick—Read a book	0.104	Austria	475	2.73	0.998	0.046	2.64	2.82
		Italy	457	2.41	0.949	0.044	2.33	2.50
		Poland	459	2.68	0.925	0.043	2.60	2.77
		Total	1391	2.61	0.968	0.026	2.56	2.66
mentally sick—Read a book	0.000	Austria	475	2.67	1.012	0.046	2.58	2.76
		Italy	457	2.40	0.891	0.042	2.32	2.48
		Poland	459	0.00	0.000	0.000	0.00	0.00
		Total	1391	1.70	1.430	0.038	1.62	1.78
physically sick—Watch TV	0.000	Austria	474	2.47	1.007	0.046	2.38	2.56
		Italy	456	2.39	0.,950	0.044	2.30	2.48
		Poland	459	2.47	0.885	0.041	2.39	2.56
		Total	1389	2.45	0.949	0.025	2.40	2.50
mentally sick—Watch TV	0.000	Austria	475	2.40	0.974	0.045	2.31	2.49
		Italy	457	2.42	0.936	0.044	2.33	2.51
		Poland	459	0.00	0.000	0.000	0.00	0.00
		Total	1391	1.61	1.376	0.037	1.54	1.69
physically sick—Sports	0.000	Austria	474	2.60	1.038	0.048	2.51	2.69
		Italy	457	2.33	0.919	0.043	2.25	2.42
		Poland	459	2.,66	0.982	0.046	2.57	2.75
		Total	1390	2.53	0.991	0.027	2.48	2.58
mentally sick—Sports	0.000	Austria	475	2.26	0.972	0.045	2.18	2.35
		Italy	457	2.21	0.892	0.042	2.13	2.30
		Poland	459	0.00	0.000	0.000	0.00	0.00
		Total	1391	1.50	1.301	.035	1.43	1.57
physically sick—Go to a park	0.000	Austria	475	2.52	0.949	0.044	2.43	2.60
		Italy	457	2.18	0.863	0.040	2.10	2.26
		Poland	459	2.59	0.871	0.041	2.51	2.67
		Total	1391	2.43	0.912	0.024	2.38	2.48
mentally sick—Go to a park	0.000	Austria	475	2.27	0.893	0.041	2.19	2.35
		Italy	457	2.17	0.837	0.039	2.09	2.24
		Poland	459	0.00	0.000	0.000	0.00	0.00
		Total	1391	1.49	1.263	0.034	1.42	1.55
physically sick—Go to a protected area	0.000	Austria	475	2.60	0.987	0.045	2.51	2.69
		Italy	457	2.25	0.789	0.037	2.18	2.32
		Poland	459	2.97	0.964	0.045	2.88	3.05
		Total	1391	2.61	0.963	0.026	2.56	2.66
mentally sick—Go to a protected area	0.000	Austria	475	2.40	0.946	0.043	2.32	2.49
		Italy	457	2.24	0.762	0.036	2.17	2.31
		Poland	459	0.00	0.000	0.000	0.00	0.00
		Total	1391	1.55	1.301	.035	1.49	1.62
physically sick—Take a walk in the city	0.281	Austria	474	2.95	0.959	0.,044	2.87	3.04
		Italy	457	2.52	0.953	0.045	2.43	2.61
		Poland	459	3.02	1.038	0.048	2.93	3.12
		Total	1390	2.83	1.008	0.027	2.78	2.89
mentally sick—Take a walk in the city	0.000	Austria	475	2.85	0.946	0.043	2.77	2.94
		Italy	457	2.52	0.948	0.044	2.43	2.61
		Poland	459	0.00	0.000	0.000	0.00	0.00
		Total	1391	1.80	1.489	0.040	1.72	1.88
physically sick—Go shopping	0.000	Austria	474	3.12	0.954	0.044	3.03	3.20
		Italy	457	2.74	0.960	0.045	2.65	2.83
		Poland	459	3.03	0.948	0.044	2.94	3.12
		Total	1390	2.96	0.967	0.026	2.91	3.01
mentally sick—Go shopping	0.000	Austria	475	3.,01	0.974	0.045	2.92	3.10
		Italy	457	2.70	0.960	0.045	2.61	2.79
		Poland	459	0.00	0.000	0.000	0.00	0.00
		Total	1391	1.92	1.566	0.042	1.83	2.00
physically sick—Listen to music	0.013	Austria	475	2.30	0.979	0.045	2.21	2.39
		Italy	457	2.12	0.909	0.043	2.03	2.20
		Poland	459	2.18	0.967	0.045	2.09	2.27
		Total	1391	2.20	0.955	0.026	2.15	2.25
mentally sick—Listen to music	0.000	Austria	475	2.13	0.928	0.043	2.05	2.21
		Italy	457	2.03	0.865	0.040	1.95	2.11
		Poland	459	0.00	0.000	0.000	0.00	0.00
		Total	1391	1.39	1.225	0.033	1.33	1.46

## Supplementary Materials

The following are available online at https://www.mdpi.com/1660-4601/16/7/1172/s1. Annex S1 Questionnaire (English translation); Annex S2 Supplementary information: Additional statistical analysis.
