# Supplementary material for: Do Protected Areas Contribute to Health and Well-Being? A Cross-Cultural Comparison
Source: Int J Environ Res Public Health. 2019 Apr 1;16(7):1172. doi: 10.3390/ijerph16071172 (PMC6479725; doi:10.3390/ijerph16071172)
Supplement: Supplementary file 1 [file ijerph-16-01172-s001.pdf]

Annex II

S2. Supplementary information: Additional statistical analysis.

Table of contents

1. Relationships between motives to visit a protected area and visitor age. ... 2

2. Relationships between motives to visit a protected area and the type of protected area ... 4

3. Relationships between the circumstances to visit a PA and visitor age ... 6

4. Relationships between the circumstances to visit a PA and the type of PA ... 8

5. Relationships between the perceived benefits of visiting a PA and the type of PA ... 11

6. Relationships between the perceived benefits of visiting a PA and visitor age ... 15

7. Relationships between the symptoms improved by stay in PA and visitor age ... 16

8. Relationships between the symptoms improved by stay in PA and the type of PA ... 19

9. Relationships between the circumstances for PA visit and country (for two visitor age categories: age < 24 years and age > 65 years ... 23

10. Relationships between the circumstances for PA visit and country (for visitor age category < 24 years) ... 24

11. Relationships between the circumstances for PA visit and country (for visitor age category > 65 years) ... 25

12. Relationships between the symptoms that can be reduced by contact with nature and country (for visitor age category < 24 years) ... 27

13. Relationships between the symptoms that can be reduced by contact with nature and country (for visitor age category > 65 years) ... 29

14. Relationships between the circumstances for PA visit and country (whole sample; results of post-hoc tests) ... 33

1. Relationships between motives to visit a protected area and visitor age.

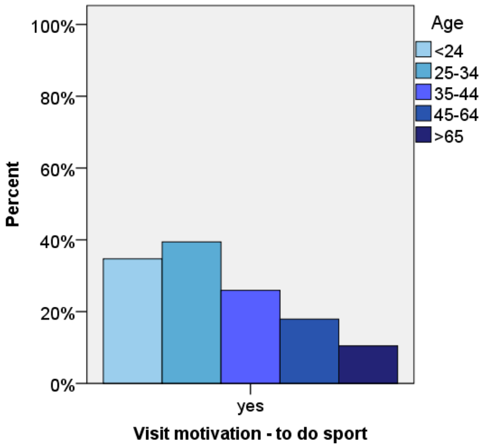

Pearson Chi-Sq =58,276; df =4; Asymp. Sig. (2-sided) = .000

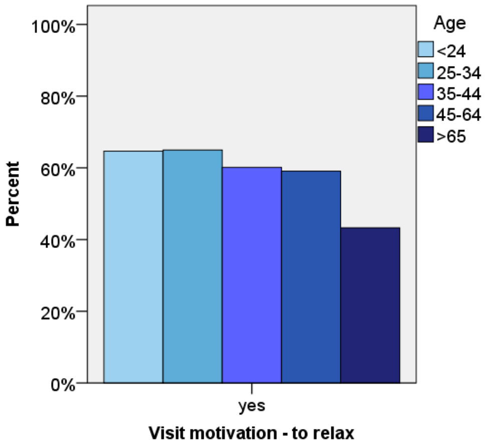

Pearson Chi-Sq = 13,455; df =4; Asymp. Sig. (2-sided) = .009

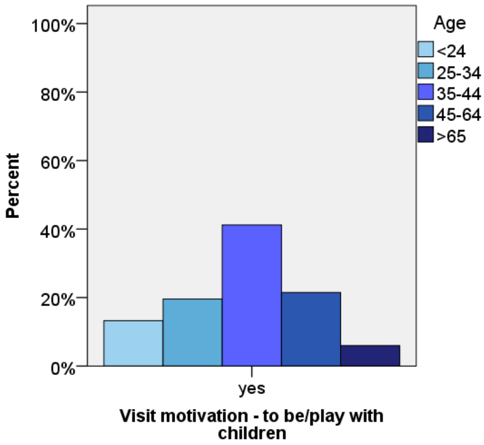

Pearson Chi-Sq = 87,66; df =4; Asymp. Sig. (2-sided) = .000

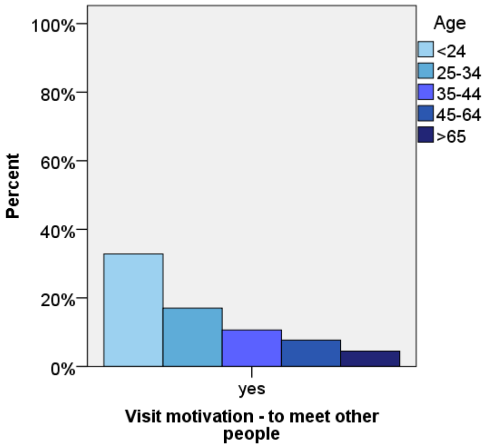

Pearson Chi-Sq =100,087; df =4; Asymp. Sig. (2-sided) =.000

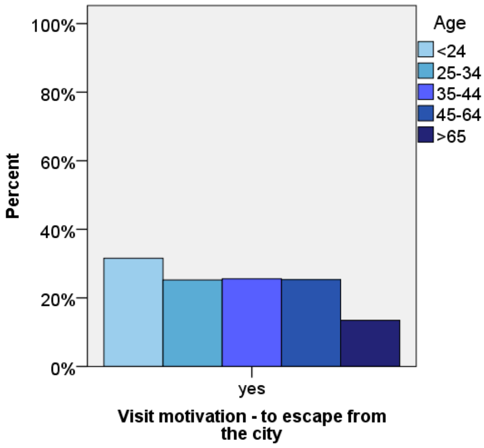

Pearson Chi-Sq =10,701; df =4; Asymp. Sig. (2-sided) = .030

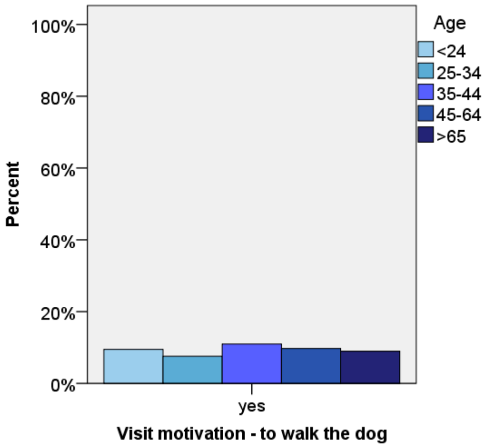

Pearson Chi-Sq = 2,172; df =4; Asymp. Sig. (2-sided) = .704

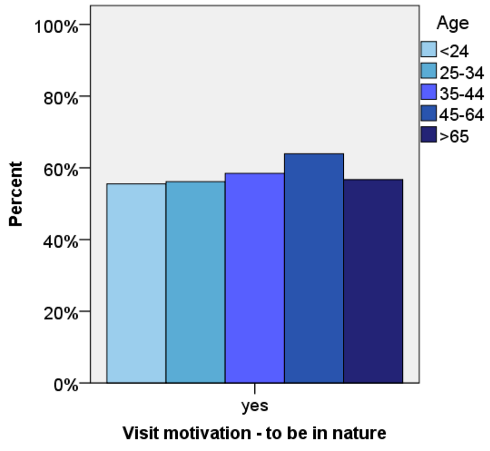

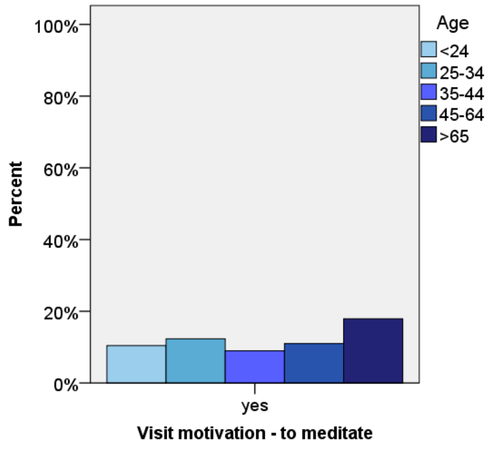

Pearson Chi-Sq = ; df =4 6,713; Asymp. Sig. (2-sided) = .152

Pearson Chi-Sq = 5,170; df =4; Asymp. Sig. (2-sided) = .270

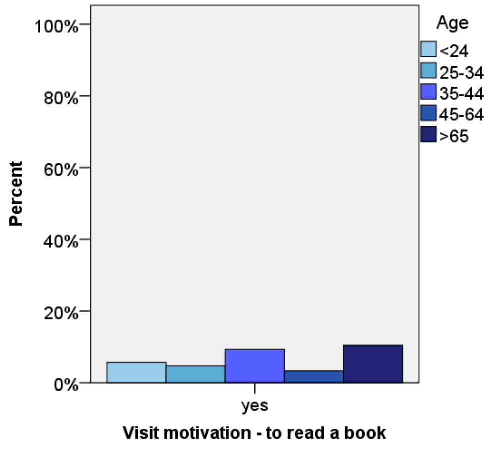

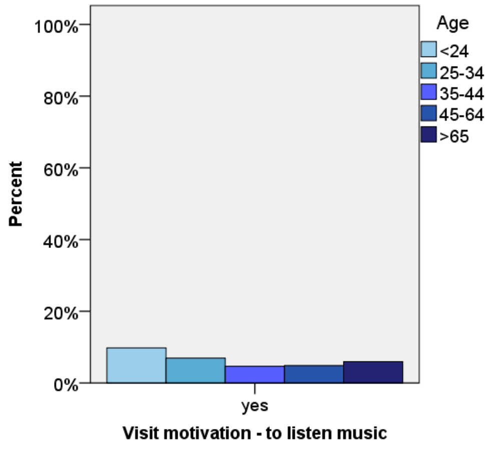

Pearson Chi-Sq = 14,427; df =4; Asymp. Sig. (2-sided) = .006

Pearson Chi-Sq = 9,214; df =4; Asymp. Sig. (2-sided) = .056

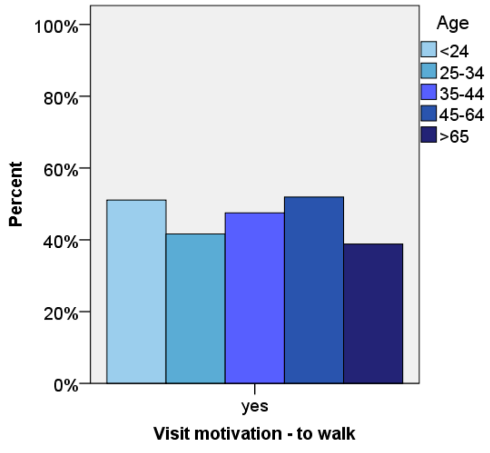

Pearson Chi-Sq = 11,047; df =4; Asymp. Sig. (2-sided) = .026

2. Relationships between motives to visit a protected area and the type of protected area

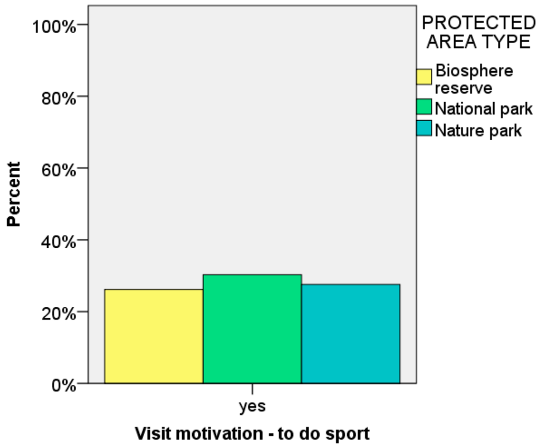

Pearson Chi-Sq = 1,997; df =2; Asymp. Sig. (2-sided) = .369

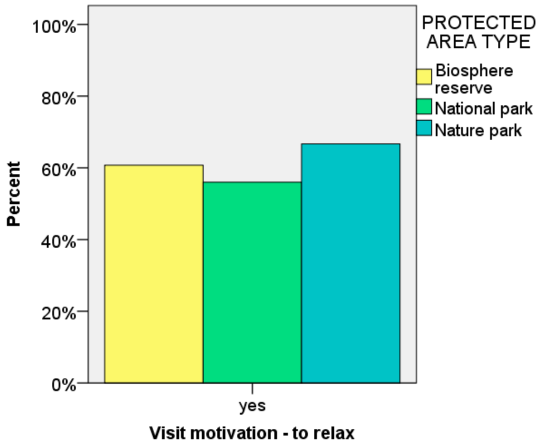

Pearson Chi-Sq = 11,173; df =2; Asymp. Sig. (2-sided) = .004

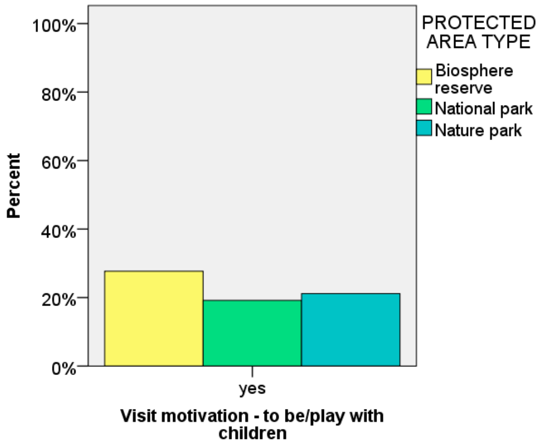

Pearson Chi-Sq = 10,491; df =2; Asymp. Sig. (2-sided) = .005

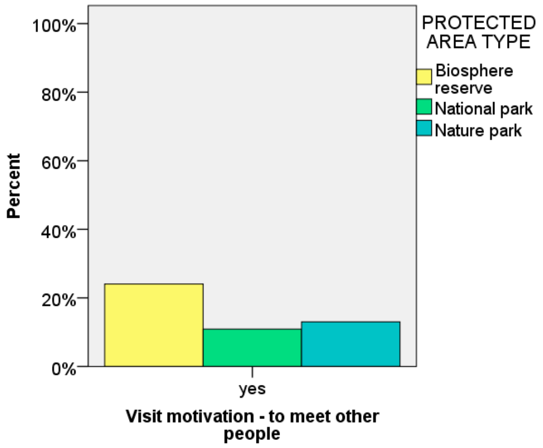

Pearson Chi-Sq = 34,336; df =2; Asymp. Sig. (2-sided) = .000

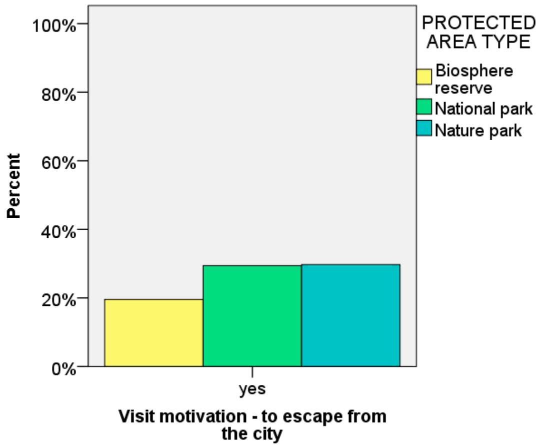

Pearson Chi-Sq = 16,143; df =2; Asymp. Sig. (2-sided) = .000

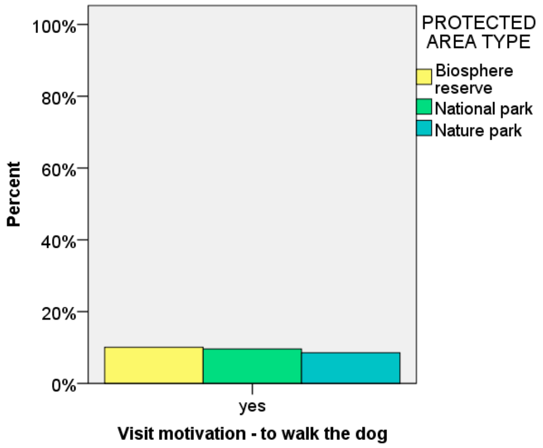

Pearson Chi-Sq = ,676; df =2; Asymp. Sig. (2-sided) = .713

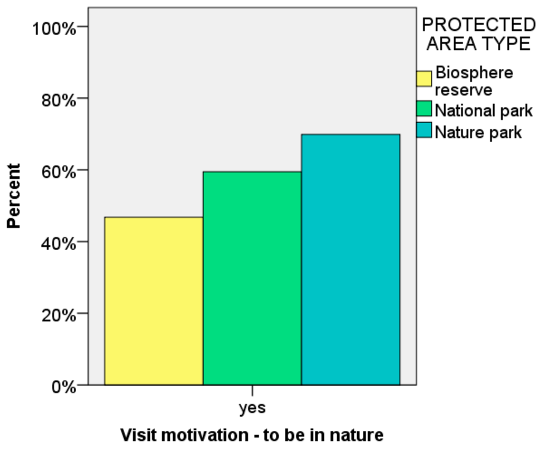

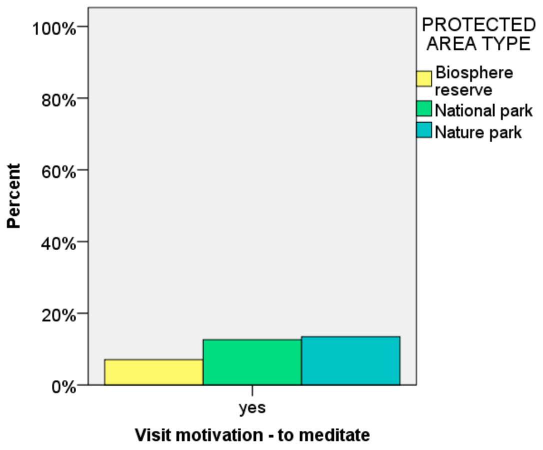

Pearson Chi-Sq = 51,523; df =2; Asymp. Sig. (2-sided) = .000

Pearson Chi-Sq =; 11,406 df =2; Asymp. Sig. (2-sided) = .003

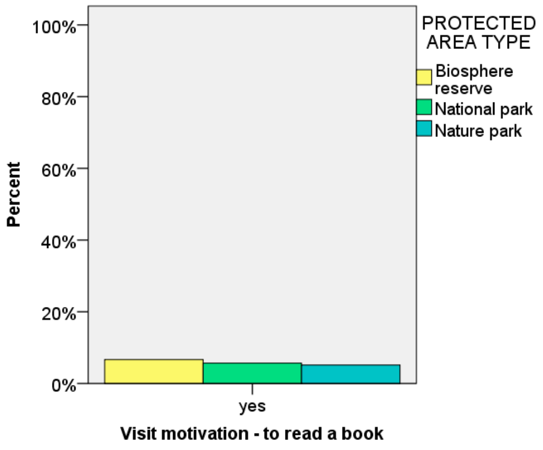

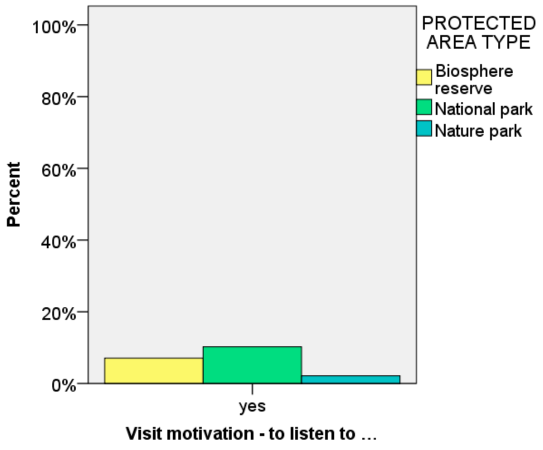

Pearson Chi-Sq = 1,019; df =2; Asymp. Sig. (2-sided) = .601

Pearson Chi-Sq = 25,622; df =2; Asymp. Sig. (2-sided) = .000

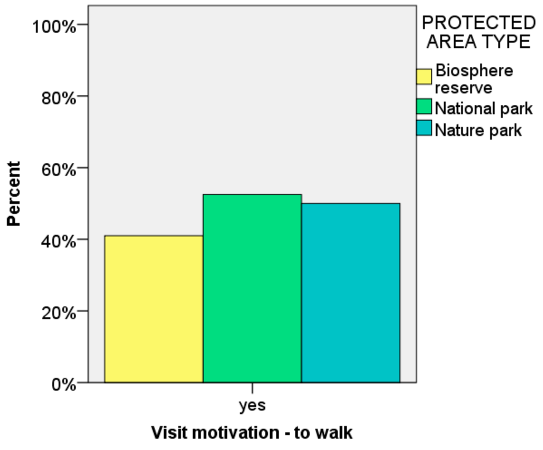

Pearson Chi-Sq = 13,649; df =2; Asymp. Sig. (2-sided) = .001

3. Relationships between the circumstances to visit a PA and visitor age

Descriptives

		95% Confidence Interval for Mean			
		Lower Bound	Upper Bound	Minimum	Maximum
Circumstances for park visit - when I need tranquility	<24	2,10	2,37	1	5
	25-34	1,89	2,11	1	5
	35-44	1,70	1,90	1	5
	45-64	1,71	1,92	1	5
	>65	1,35	1,66	1	3
	Total	1,88	1,99	1	5
Circumstances for park visit - when I'm feeling good	<24	1,97	2,20	1	5
	25-34	1,93	2,12	1	5
	35-44	1,75	1,95	1	5
	45-64	1,82	1,98	1	5
	>65	1,55	1,94	1	5
	Total	1,91	2,00	1	5
Circumstances for park visit - when I'm feeling bad (emotionally stressed, or depressed)	<24	2,69	2,97	1	5
	25-34	2,38	2,63	1	5
	35-44	2,62	2,90	1	5
	45-64	2,40	2,65	1	5
	>65	2,34	2,92	1	5
	Total	2,58	2,71	1	5
Circumstances for park visit - when I want to escape from the city	<24	2,23	2,52	1	5
	25-34	1,97	2,24	1	5
	35-44	1,78	2,04	1	5
	45-64	2,01	2,28	1	5
	>65	1,58	2,01	1	5
	Total	2,06	2,19	1	5

ANOVA

		Sum of Squares	df	Mean Square	F
Circumstances for park visit - when I need tranquility	Between Groups	53,432	4	13,358	12,512
	Within Groups	1480,749	1387	1,068	
	Total	1534,181	1391		
Circumstances for park visit - when I'm feeling good	Between Groups	14,313	4	3,578	4,534
	Within Groups	1094,557	1387	,789	
	Total	1108,871	1391		
Circumstances for park visit - when I'm feeling bad (emotionally stressed, or depressed)	Between Groups	27,308	4	6,827	4,587
	Within Groups	2064,502	1387	1,488	
	Total	2091,810	1391		

Circumstances for park visit - when I want to escape from the city	Between Groups	41,509	4	10,377	6,782
	Within Groups	2122,215	1387	1,530	
	Total	2163,724	1391		

ANOVA

		Sig.
Circumstances for park visit - when I need tranquility	Between Groups	,000
	Within Groups	
	Total	
Circumstances for park visit - when I'm feeling good	Between Groups	,001
	Within Groups	
	Total	
Circumstances for park visit - when I'm feeling bad (emotionally stressed, or depressed)	Between Groups	,001
	Within Groups	
	Total	
Circumstances for park visit - when I want to escape from the city	Between Groups	,000
	Within Groups	
	Total	

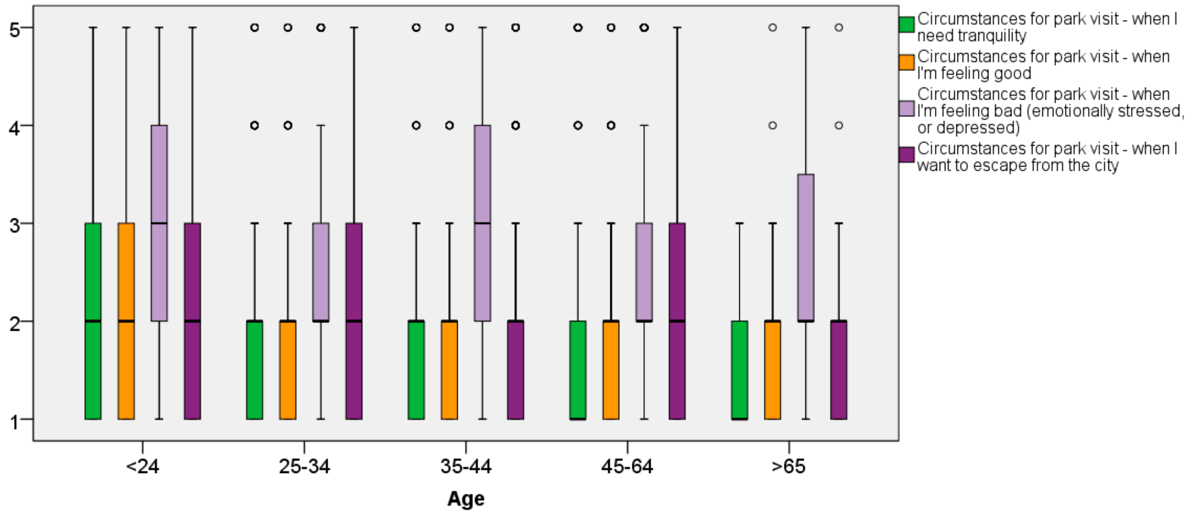

4. Relationships between the circumstances to visit a PA and the type of PA

Descriptives

		N	Mean	Std. Deviation	Std. Error
Circumstances for park visit - when I need tranquility	Biosphere reserve	466	2,20	1,234	,057
	National park	459	1,91	,914	,043
	Nature park	467	1,70	,910	,042
	Total	1392	1,94	1,050	,028
Circumstances for park visit - when I'm feeling good	Biosphere reserve	466	2,09	,995	,046
	National park	459	1,97	,853	,040
	Nature park	467	1,80	,797	,037
	Total	1392	1,95	,893	,024
Circumstances for park visit - when I'm feeling bad (emotionally stressed, or depressed)	Biosphere reserve	466	2,79	1,298	,060
	National park	459	2,79	1,209	,056
	Nature park	467	2,36	1,118	,052
	Total	1392	2,65	1,226	,033
Circumstances for park visit - when I want to escape from the city	Biosphere reserve	466	2,34	1,362	,063
	National park	459	2,07	1,172	,055
	Nature park	467	1,95	1,167	,054
	Total	1392	2,12	1,247	,033

Descriptives

		95% Confidence Interval for Mean		
		Lower Bound	Upper Bound	Minimum
Circumstances for park visit - when I need tranquility	Biosphere reserve	2,09	2,31	1
	National park	1,82	1,99	1
	Nature park	1,62	1,79	1
	Total	1,88	1,99	1
Circumstances for park visit - when I'm feeling good	Biosphere reserve	2,00	2,18	1
	National park	1,89	2,05	1
	Nature park	1,73	1,88	1
	Total	1,91	2,00	1
Circumstances for park visit - when I'm feeling bad (emotionally stressed, or depressed)	Biosphere reserve	2,67	2,91	1
	National park	2,68	2,90	1
	Nature park	2,26	2,47	1
	Total	2,58	2,71	1
Circumstances for park visit - when I want to escape from the city	Biosphere reserve	2,22	2,47	1
	National park	1,96	2,18	1
	Nature park	1,84	2,05	1
	Total	2,06	2,19	1

Descriptives

		Maximum
Circumstances for park visit - when I need tranquility	Biosphere reserve	5
	National park	5
	Nature park	5
	Total	5
Circumstances for park visit - when I'm feeling good	Biosphere reserve	5
	National park	5
	Nature park	5
	Total	5
Circumstances for park visit - when I'm feeling bad (emotionally stressed, or depressed)	Biosphere reserve	5
	National park	5
	Nature park	5
	Total	5
Circumstances for park visit - when I want to escape from the city	Biosphere reserve	5
	National park	5
	Nature park	5
	Total	5

ANOVA

		Sum of Squares	df	Mean Square	F
Circumstances for park visit - when I need tranquility	Between Groups	57,745	2	28,873	27,163
	Within Groups	1476,436	1389	1,063	
	Total	1534,181	1391		
Circumstances for park visit - when I'm feeling good	Between Groups	18,855	2	9,428	12,014
	Within Groups	1090,015	1389	,785	
	Total	1108,871	1391		
Circumstances for park visit - when I'm feeling bad (emotionally stressed, or depressed)	Between Groups	56,382	2	28,191	19,238
	Within Groups	2035,428	1389	1,465	
	Total	2091,810	1391		
Circumstances for park visit - when I want to escape from the city	Between Groups	37,581	2	18,791	12,276
	Within Groups	2126,143	1389	1,531	
	Total	2163,724	1391		

ANOVA

		Sig.
Circumstances for park visit - when I need tranquility	Between Groups	,000
	Within Groups	
	Total	

Circumstances for park visit - when I'm feeling good	Between Groups	,000
	Within Groups	
	Total	
Circumstances for park visit - when I'm feeling bad (emotionally stressed, or depressed)	Between Groups	,000
	Within Groups	
	Total	
Circumstances for park visit - when I want to escape from the city	Between Groups	,000
	Within Groups	
	Total	

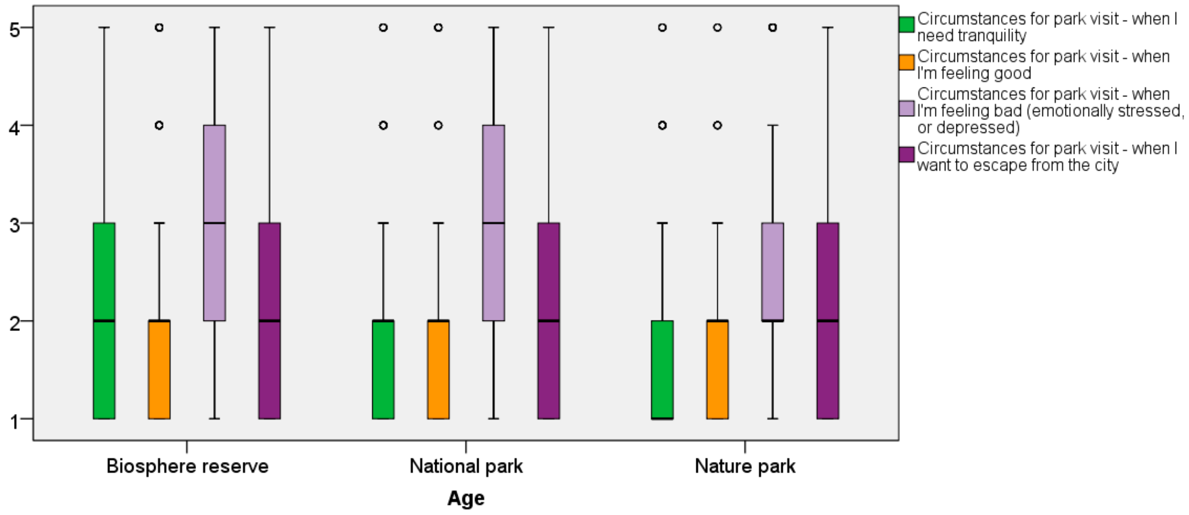

5. Relationships between the perceived benefits of visiting a PA and the type of PA

Descriptives

		N	Mean	Std. Deviation	Std. Error	95% Confidence Interval for Mean Lower Bound	95% Confidence Interval for Mean Upper Bound	Minimum
in this park there is a very healthy air quality	Biosphere reserve	466	1,67	,727	,034	1,60	1,73	1
	National park	459	1,55	,732	,034	1,48	1,62	1
	Nature park	467	1,62	,841	,039	1,54	1,70	1
	Total	1392	1,61	,770	,021	1,57	1,65	1
wild nature makes me feel happy	Biosphere reserve	466	1,68	,758	,035	1,61	1,75	1
	National park	459	1,65	,812	,038	1,58	1,73	1
	Nature park	467	1,56	,771	,036	1,49	1,63	1
	Total	1392	1,63	,781	,021	1,59	1,67	1
in protected areas the pollution is low	Biosphere reserve	466	2,00	,860	,040	1,92	2,07	1
	National park	459	2,05	,895	,042	1,97	2,13	1
	Nature park	467	1,93	,916	,042	1,85	2,01	1
	Total	1392	1,99	,891	,024	1,94	2,04	1
to stay in contact with nature let's me forget my daily worries	Biosphere reserve	466	2,02	,917	,042	1,94	2,10	1
	National park	459	1,97	,925	,043	1,89	2,06	1
	Nature park	467	1,95	,924	,043	1,86	2,03	1
	Total	1392	1,98	,922	,025	1,93	2,03	1
a stay in a potected area increases my wellbeing	Biosphere reserve	466	1,92	,880	,041	1,84	2,00	1
	National park	459	1,83	,901	,042	1,75	1,91	1
	Nature park	467	1,76	,894	,041	1,68	1,84	1
	Total	1392	1,84	,893	,024	1,79	1,88	1
wild nature makes me feel free	Biosphere reserve	466	1,98	,933	,043	1,90	2,07	1
	National park	459	2,02	1,088	,051	1,92	2,12	1
	Nature park	467	1,91	1,025	,047	1,82	2,00	1
	Total	1392	1,97	1,017	,027	1,92	2,02	1
in wild nature i am anxious, because i get lost	Biosphere reserve	466	3,65	1,255	,058	3,53	3,76	1
	National park	459	3,67	1,262	,059	3,55	3,78	1
	Nature park	467	3,57	1,310	,061	3,45	3,69	1
	Total	1392	3,63	1,276	,034	3,56	3,69	1
a stay in this park is healthy for me	Biosphere reserve	466	1,75	,782	,036	1,68	1,82	1
	National park	459	1,77	,849	,040	1,69	1,84	1
	Nature park	467	1,80	,906	,042	1,71	1,88	1
	Total	1392	1,77	,847	,023	1,73	1,82	1
protected areas are important for personal wellbeing	Biosphere reserve	466	1,93	,864	,040	1,85	2,01	1
	National park	459	1,87	,863	,040	1,79	1,95	1
	Nature park	467	1,84	,866	,040	1,76	1,91	1
	Total	1392	1,88	,865	,023	1,83	1,92	1

a stay in nature makes me feel relaxed (Formulierung vergleichen)	Biosphere reserve	466	1,75	,737	,034	1,69	1,82	1
	National park	459	1,71	,783	,037	1,64	1,78	1
	Nature park	467	1,64	,768	,036	1,57	1,71	1
	Total	1392	1,70	,764	,020	1,66	1,74	1
to stay in contact with nature recharges my battery	Biosphere reserve	466	1,76	,848	,039	1,69	1,84	1
	National park	459	1,79	,824	,038	1,72	1,87	1
	Nature park	467	1,73	,850	,039	1,66	1,81	1
	Total	1392	1,76	,841	,023	1,72	1,81	1
to stay in contact with nature restores my pshychophysical equilibrium	Biosphere reserve	466	1,87	,869	,040	1,79	1,95	1
	National park	459	1,92	,894	,042	1,83	2,00	1
	Nature park	467	1,81	,863	,040	1,73	1,89	1
	Total	1392	1,87	,876	,023	1,82	1,91	1

Descriptives

		Maximum
in this park there is a very healthy air quality	Biosphere reserve	4
	National park	5
	Nature park	5
	Total	5
wild nature makes me feel happy	Biosphere reserve	5
	National park	5
	Nature park	5
	Total	5
in protected areas the pollution is low	Biosphere reserve	5
	National park	5
	Nature park	5
	Total	5
to stay in contact with nature let's me forget my daily worries	Biosphere reserve	5
	National park	5
	Nature park	5
	Total	5
a stay in a potected area increases my wellbeing	Biosphere reserve	5
	National park	5
	Nature park	5
	Total	5
wild nature makes me feel free	Biosphere reserve	5
	National park	5
	Nature park	5
	Total	5
in wild nature i am anxious, because i get lost	Biosphere reserve	5
	National park	5
	Nature park	5

	Total	5
a stay in this park is healthy for me	Biosphere reserve	5
	National park	5
	Nature park	5
	Total	5
protected areas are important for personal wellbeing	Biosphere reserve	5
	National park	5
	Nature park	5
	Total	5
a stay in nature makes me feel relaxed (Formulierung vergleichen)	Biosphere reserve	5
	National park	5
	Nature park	5
	Total	5
to stay in contact with nature recharges my battery	Biosphere reserve	5
	National park	5
	Nature park	5
	Total	5
to stay in contact with nature restores my pshychophysical equilibrium	Biosphere reserve	5
	National park	5
	Nature park	5
	Total	5

ANOVA

		Sum of Squares	df	Mean Square	F	Sig.
in this park there is a very healthy air quality	Between Groups	3,277	2	1,639	2,773	,063
	Within Groups	821,390	1390	,591		
	Total	824,668	1392			
wild nature makes me feel happy	Between Groups	3,835	2	1,918	3,150	,043
	Within Groups	846,242	1390	,609		
	Total	850,078	1392			
in protected areas the pollution is low	Between Groups	3,148	2	1,574	1,984	,138
	Within Groups	1102,749	1390	,793		
	Total	1105,897	1392			
to stay in contact with nature let's me forget my daily worries	Between Groups	1,195	2	,597	,703	,495
	Within Groups	1181,282	1390	,850		
	Total	1182,477	1392			
a stay in a potected area increases my wellbeing	Between Groups	6,020	2	3,010	3,788	,023
	Within Groups	1104,662	1390	,795		
	Total	1110,682	1392			
wild nature makes me feel free	Between Groups	2,954	2	1,477	1,429	,240
	Within Groups	1436,839	1390	1,034		

	Total	1439,793	1392			
in wild nature i am anxious, because i get lost	Between Groups	2,698	2	1,349	,829	,437
	Within Groups	2263,188	1390	1,628		
	Total	2265,887	1392			
a stay in this park is healthy for me	Between Groups	,562	2	,281	,391	,676
	Within Groups	997,334	1389	,718		
	Total	997,896	1391			
protected areas are important for personal wellbeing	Between Groups	2,019	2	1,009	1,351	,259
	Within Groups	1038,744	1390	,747		
	Total	1040,762	1392			
a stay in nature makes me feel relaxed (Formulierung vergleichen)	Between Groups	2,977	2	1,488	2,557	,078
	Within Groups	809,193	1390	,582		
	Total	812,169	1392			
to stay in contact with nature recharges my battery	Between Groups	,838	2	,419	,593	,553
	Within Groups	982,985	1390	,707		
	Total	983,823	1392			
to stay in contact with nature restores my pshychophysical equilibrium	Between Groups	2,495	2	1,248	1,629	,197
	Within Groups	1064,669	1390	,766		
	Total	1067,164	1392			

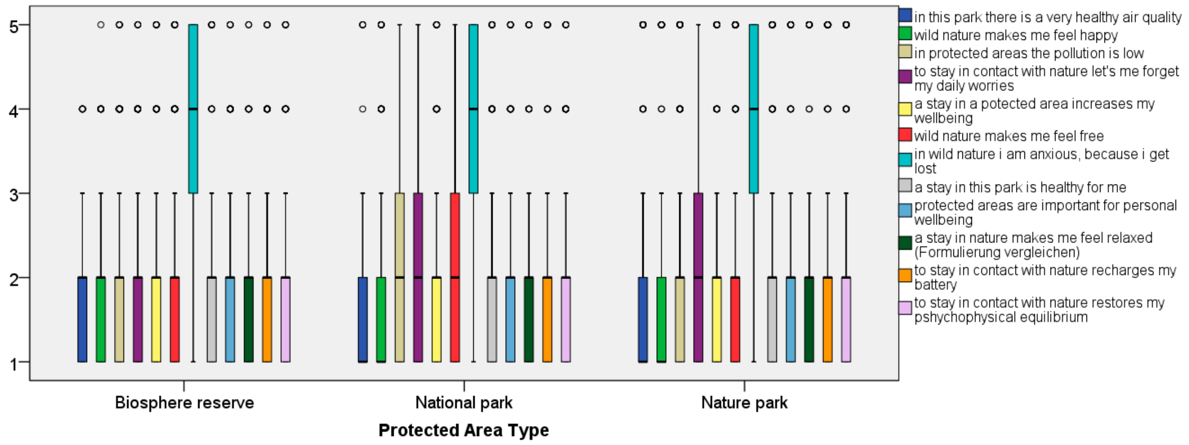

6. Relationships between the perceived benefits of visiting a PA and visitor age

ANOVA

		Sum of Squares	df	Mean Square	F	Sig.
in this park there is a very healthy air quality	Between Groups	8,009	4	2,002	3,403	,009
	Within Groups	816,659	1388	,588		
	Total	824,668	1392			
wild nature makes me feel happy	Between Groups	7,153	4	1,788	2,945	,019
	Within Groups	842,925	1388	,607		
	Total	850,078	1392			
in protected areas the pollution is low	Between Groups	2,938	4	,734	,924	,449
	Within Groups	1102,959	1388	,795		
	Total	1105,897	1392			
to stay in contact with nature let's me forget my daily worries	Between Groups	31,931	4	7,983	9,630	,000
	Within Groups	1150,546	1388	,829		
	Total	1182,477	1392			
a stay in a potected area increases my wellbeing	Between Groups	32,665	4	8,166	10,514	,000
	Within Groups	1078,017	1388	,777		
	Total	1110,682	1392			
wild nature makes me feel free	Between Groups	21,046	4	5,262	5,148	,000
	Within Groups	1418,747	1388	1,022		
	Total	1439,793	1392			
in wild nature i am anxious, because i get lost	Between Groups	16,407	4	4,102	2,531	,039
	Within Groups	2249,480	1388	1,621		
	Total	2265,887	1392			
a stay in this park is healthy for me	Between Groups	3,916	4	,979	1,366	,243
	Within Groups	993,979	1387	,717		
	Total	997,896	1391			
protected areas are important for personal wellbeing	Between Groups	28,158	4	7,040	9,649	,000
	Within Groups	1012,604	1388	,730		
	Total	1040,762	1392			
a stay in nature makes me feel relaxed (Formulierung vergleichen)	Between Groups	8,722	4	2,181	3,767	,005
	Within Groups	803,447	1388	,579		
	Total	812,169	1392			
to stay in contact with nature recharges my battery	Between Groups	20,842	4	5,210	7,510	,000
	Within Groups	962,982	1388	,694		
	Total	983,823	1392			
to stay in contact with nature restores my pshychophysical equilibrium	Between Groups	24,016	4	6,004	7,989	,000
	Within Groups	1043,149	1388	,752		
	Total	1067,164	1392			

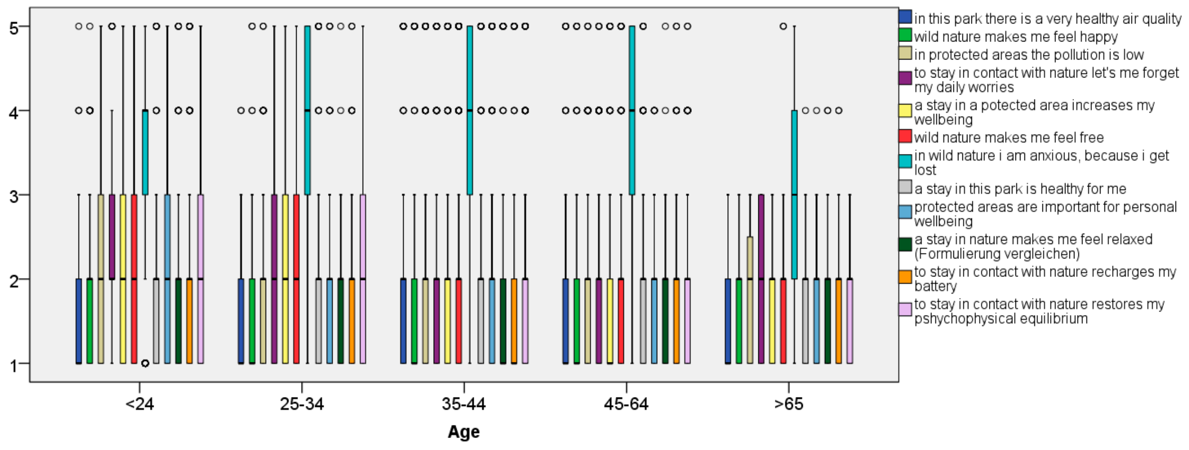

7. Relationships between the symptoms improved by stay in PA and visitor age

Descriptives

		N	Mean	Std. Deviation	Std. Error	95% Confidence Interval for Mean Lower Bound	Upper Bound	Minimum	Maximum
Symptoms that can be reduced by contact with nature - Iritability	<24	316	1,87	,826	,046	1,78	1,96	1	5
	25-34	316	1,79	,762	,043	1,70	1,87	1	5
	35-44	300	1,58	,725	,042	1,50	1,66	1	5
	45-64	391	1,56	,680	,034	1,49	1,63	1	4
	>65	67	1,67	,705	,086	1,50	1,84	1	3
	Total	1390	1,69	,755	,020	1,65	1,73	1	5
Symptoms that can be reduced by contact with nature - Lack of ability to concentrate	<24	316	2,17	,913	,051	2,07	2,27	1	5
	25-34	316	1,96	,786	,044	1,87	2,05	1	5
	35-44	300	1,91	,899	,052	1,80	2,01	1	5
	45-64	391	1,85	,848	,043	1,77	1,94	1	5
	>65	67	1,91	,668	,082	1,75	2,07	1	3
	Total	1390	1,96	,860	,023	1,92	2,01	1	5
Symptoms that can be reduced by contact with nature - Insomnia	<24	316	2,46	,923	,052	2,36	2,56	1	5
	25-34	316	2,21	,846	,048	2,11	2,30	1	5
	35-44	301	2,15	1,018	,059	2,04	2,27	1	5
	45-64	390	2,05	,964	,049	1,95	2,14	1	5
	>65	67	2,21	,686	,084	2,04	2,38	1	4
	Total	1390	2,21	,941	,025	2,16	2,26	1	5
Symptoms that can be reduced by contact with nature - Muscolar tension	<24	316	2,82	1,093	,061	2,70	2,94	1	5
	25-34	313	2,39	,917	,052	2,28	2,49	1	5
	35-44	301	2,32	1,023	,059	2,21	2,44	1	5
	45-64	387	2,19	,951	,048	2,09	2,28	1	5
	>65	67	2,07	,804	,098	1,88	2,27	1	4
	Total	1384	2,40	1,016	,027	2,35	2,46	1	5

Symptoms that can be reduced by contact with nature - Pain	<24	316	2,70	1,015	,057	2,59	2,81	1	5
	25-34	316	2,49	,934	,053	2,39	2,60	1	5
	35-44	301	2,43	1,036	,060	2,31	2,55	1	5
	45-64	388	2,39	1,084	,055	2,29	2,50	1	5
	>65	67	2,70	,985	,120	2,46	2,94	1	5
	Total	1388	2,51	1,026	,028	2,46	2,56	1	5
Symptoms that can be reduced by contact with nature - General anxiety	<24	315	2,23	,942	,053	2,13	2,34	1	5
	25-34	316	2,06	,905	,051	1,96	2,16	1	5
	35-44	301	1,97	,923	,053	1,87	2,08	1	5
	45-64	390	1,89	,856	,043	1,80	1,97	1	5
	>65	67	1,84	,593	,072	1,69	1,98	1	3
	Total	1389	2,02	,900	,024	1,97	2,07	1	5
Symptoms that can be reduced by contact with nature - Depression	<24	316	2,05	,958	,054	1,94	2,16	1	5
	25-34	316	2,15	,995	,056	2,04	2,26	1	5
	35-44	301	1,98	,995	,057	1,87	2,10	1	5
	45-64	391	2,06	1,042	,053	1,96	2,17	1	5
	>65	67	2,10	,761	,093	1,92	2,29	1	4
	Total	1391	2,06	,991	,027	2,01	2,12	1	5
Symptoms that can be reduced by contact with nature - Short term memory	<24	317	2,96	1,103	,062	2,84	3,08	1	5
	25-34	316	2,76	1,096	,062	2,64	2,88	1	5
	35-44	301	2,73	1,098	,063	2,60	2,85	1	5
	45-64	386	2,55	1,143	,058	2,44	2,67	1	5
	>65	67	2,75	,990	,121	2,50	2,99	1	5
	Total	1387	2,74	1,114	,030	2,68	2,80	1	5

ANOVA

		Sum of Squares	df	Mean Square	F
Symptoms that can be reduced by contact with nature - Iritability	Between Groups	23,903	4	5,976	10,778
	Within Groups	767,925	1385	,554	
	Total	791,828	1389		
Symptoms that can be reduced by contact with nature - Lack of ability to concentrate	Between Groups	19,424	4	4,856	6,667
	Within Groups	1008,777	1385	,728	
	Total	1028,201	1389		
Symptoms that can be reduced by contact with nature - Insomnia	Between Groups	31,525	4	7,881	9,116
	Within Groups	1197,388	1385	,865	
	Total	1228,913	1389		
Symptoms that can be reduced by contact with nature - Muscolar tension	Between Groups	82,739	4	20,685	21,225
	Within Groups	1343,897	1379	,975	
	Total	1426,636	1383		
Symptoms that can be reduced by contact with nature - Pain	Between Groups	20,900	4	5,225	5,018
	Within Groups	1439,978	1383	1,041	

	Total	1460,878	1387		
Symptoms that can be reduced by contact with nature - General anxiety	Between Groups	24,861	4	6,215	7,817
	Within Groups	1100,491	1384	,795	
	Total	1125,352	1388		
Symptoms that can be reduced by contact with nature - Depression	Between Groups	4,390	4	1,098	1,119
	Within Groups	1359,787	1386	,981	
	Total	1364,177	1390		
Symptoms that can be reduced by contact with nature - Short term memory	Between Groups	28,632	4	7,158	5,845
	Within Groups	1692,409	1382	1,225	
	Total	1721,041	1386		

ANOVA

		Sig.
Symptoms that can be reduced by contact with nature - Iritability	Between Groups	,000
	Within Groups	
	Total	
Symptoms that can be reduced by contact with nature - Lack of ability to concentrate	Between Groups	,000
	Within Groups	
	Total	
Symptoms that can be reduced by contact with nature - Insomnia	Between Groups	,000
	Within Groups	
	Total	
Symptoms that can be reduced by contact with nature - Muscolar tension	Between Groups	,000
	Within Groups	
	Total	
Symptoms that can be reduced by contact with nature - Pain	Between Groups	,001
	Within Groups	
	Total	
Symptoms that can be reduced by contact with nature - General anxiety	Between Groups	,000
	Within Groups	
	Total	
Symptoms that can be reduced by contact with nature - Depression	Between Groups	,346
	Within Groups	
	Total	
Symptoms that can be reduced by contact with nature - Short term memory	Between Groups	,000
	Within Groups	
	Total	

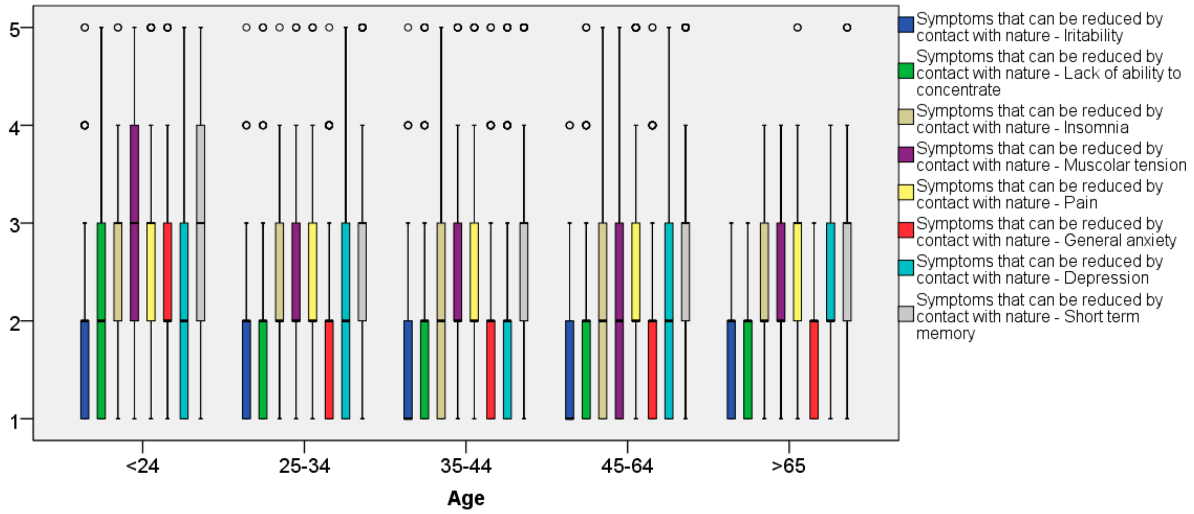

8. Relationships between the symptoms improved by stay in PA and the type of PA

Descriptives

		N	Mean	Std. Deviation	Std. Error
Symptoms that can be reduced by contact with nature - Iritability	Biosphere reserve	465	1,78	,843	,039
	National park	459	1,73	,734	,034
	Nature park	466	1,57	,663	,031
	Total	1390	1,69	,755	,020
Symptoms that can be reduced by contact with nature - Lack of ability to concentrate	Biosphere reserve	464	2,03	,868	,040
	National park	459	1,98	,865	,040
	Nature park	467	1,88	,844	,039
	Total	1390	1,96	,860	,023
Symptoms that can be reduced by contact with nature - Insomnia	Biosphere reserve	464	2,30	,956	,044
	National park	459	2,21	,934	,044
	Nature park	467	2,12	,925	,043
	Total	1390	2,21	,941	,025
Symptoms that can be reduced by contact with nature - Muscolar tension	Biosphere reserve	462	2,42	1,015	,047
	National park	459	2,52	1,012	,047
	Nature park	463	2,27	1,008	,047
	Total	1384	2,40	1,016	,027
Symptoms that can be reduced by contact with nature - Pain	Biosphere reserve	462	2,57	1,074	,050
	National park	459	2,57	1,005	,047
	Nature park	467	2,39	,991	,046
	Total	1388	2,51	1,026	,028
Symptoms that can be reduced by contact with nature - General anxiety	Biosphere reserve	463	2,07	,948	,044
	National park	459	2,12	,922	,043
	Nature park	467	1,88	,811	,038
	Total	1389	2,02	,900	,024
Symptoms that can be reduced by	Biosphere reserve	465	2,13	1,058	,049

contact with nature - Depression	National park	459	2,11	,943	,044
	Nature park	467	1,95	,959	,044
	Total	1391	2,06	,991	,027
Symptoms that can be reduced by contact with nature - Short term memory	Biosphere reserve	464	2,71	1,069	,050
	National park	459	2,89	1,142	,053
	Nature park	464	2,62	1,115	,052
	Total	1387	2,74	1,114	,030

Descriptives

		95% Confidence Interval for Mean			
		Lower Bound	Upper Bound	Minimum	Maximum
Symptoms that can be reduced by contact with nature - Iritability	Biosphere reserve	1,70	1,86	1	5
	National park	1,66	1,80	1	4
	Nature park	1,51	1,63	1	5
	Total	1,65	1,73	1	5
Symptoms that can be reduced by contact with nature - Lack of ability to concentrate	Biosphere reserve	1,95	2,11	1	5
	National park	1,90	2,06	1	5
	Nature park	1,81	1,96	1	5
	Total	1,92	2,01	1	5
Symptoms that can be reduced by contact with nature - Insomnia	Biosphere reserve	2,21	2,39	1	5
	National park	2,12	2,29	1	5
	Nature park	2,03	2,20	1	5
	Total	2,16	2,26	1	5
Symptoms that can be reduced by contact with nature - Muscolar tension	Biosphere reserve	2,33	2,51	1	5
	National park	2,42	2,61	1	5
	Nature park	2,18	2,36	1	5
	Total	2,35	2,46	1	5
Symptoms that can be reduced by contact with nature - Pain	Biosphere reserve	2,47	2,67	1	5
	National park	2,48	2,66	1	5
	Nature park	2,30	2,48	1	5
	Total	2,46	2,56	1	5
Symptoms that can be reduced by contact with nature - General anxiety	Biosphere reserve	1,98	2,15	1	5
	National park	2,03	2,20	1	5
	Nature park	1,81	1,96	1	5
	Total	1,97	2,07	1	5
Symptoms that can be reduced by contact with nature - Depression	Biosphere reserve	2,03	2,23	1	5
	National park	2,02	2,20	1	5
	Nature park	1,87	2,04	1	5
	Total	2,01	2,12	1	5
Symptoms that can be reduced by contact with nature - Short term memory	Biosphere reserve	2,61	2,81	1	5
	National park	2,79	3,00	1	5
	Nature park	2,51	2,72	1	5
	Total	2,68	2,80	1	5

ANOVA

		Sum of Squares	df	Mean Square	F
Symptoms that can be reduced by contact with nature - Iritability	Between Groups	11,399	2	5,699	10,129
	Within Groups	780,429	1387	,563	
	Total	791,828	1389		
Symptoms that can be reduced by contact with nature - Lack of ability to concentrate	Between Groups	4,986	2	2,493	3,380
	Within Groups	1023,215	1387	,738	
	Total	1028,201	1389		
Symptoms that can be reduced by contact with nature - Insomnia	Between Groups	7,693	2	3,846	4,369
	Within Groups	1221,220	1387	,880	
	Total	1228,913	1389		
Symptoms that can be reduced by contact with nature - Muscolar tension	Between Groups	14,219	2	7,109	6,951
	Within Groups	1412,417	1381	1,023	
	Total	1426,636	1383		
Symptoms that can be reduced by contact with nature - Pain	Between Groups	9,717	2	4,859	4,637
	Within Groups	1451,161	1385	1,048	
	Total	1460,878	1387		
Symptoms that can be reduced by contact with nature - General anxiety	Between Groups	14,258	2	7,129	8,893
	Within Groups	1111,094	1386	,802	
	Total	1125,352	1388		
Symptoms that can be reduced by contact with nature - Depression	Between Groups	8,882	2	4,441	4,548
	Within Groups	1355,295	1388	,976	
	Total	1364,177	1390		
Symptoms that can be reduced by contact with nature - Short term memory	Between Groups	18,255	2	9,127	7,419
	Within Groups	1702,786	1384	1,230	
	Total	1721,041	1386		

ANOVA

		Sig.
Symptoms that can be reduced by contact with nature - Iritability	Between Groups	,000
	Within Groups	
	Total	
Symptoms that can be reduced by contact with nature - Lack of ability to concentrate	Between Groups	,034
	Within Groups	
	Total	
Symptoms that can be reduced by contact with nature - Insomnia	Between Groups	,013
	Within Groups	
	Total	
Symptoms that can be reduced by contact with nature - Muscolar tension	Between Groups	,001
	Within Groups	

	Total	
Symptoms that can be reduced by contact with nature - Pain	Between Groups	,010
	Within Groups	
	Total	
Symptoms that can be reduced by contact with nature - General anxiety	Between Groups	,000
	Within Groups	
	Total	
Symptoms that can be reduced by contact with nature - Depression	Between Groups	,011
	Within Groups	
	Total	
Symptoms that can be reduced by contact with nature - Short term memory	Between Groups	,001
	Within Groups	
	Total	

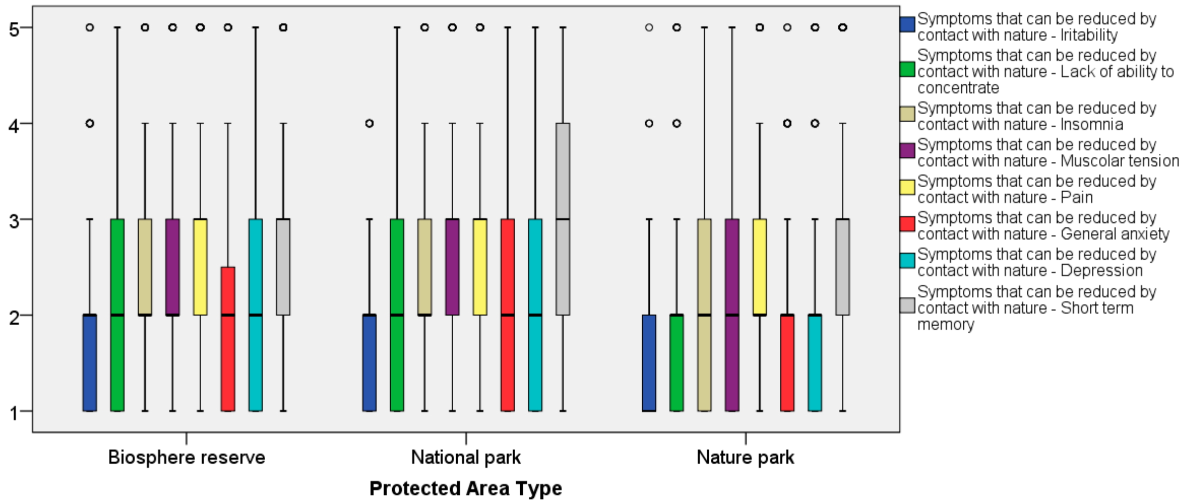

9. Relationships between the circumstances for PA visit and country (for two visitor age categories: age < 24 years and age > 65 years

Means Plots

Age < 24 years	Age > 65 years

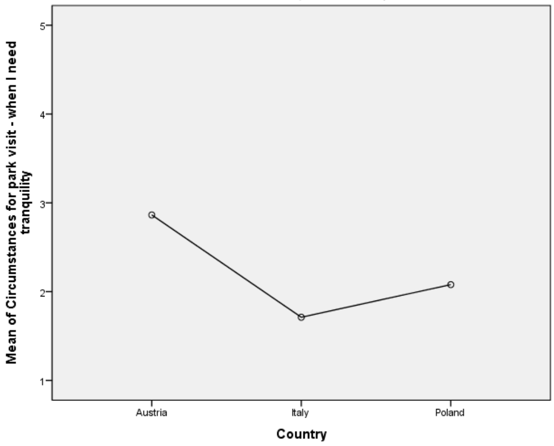

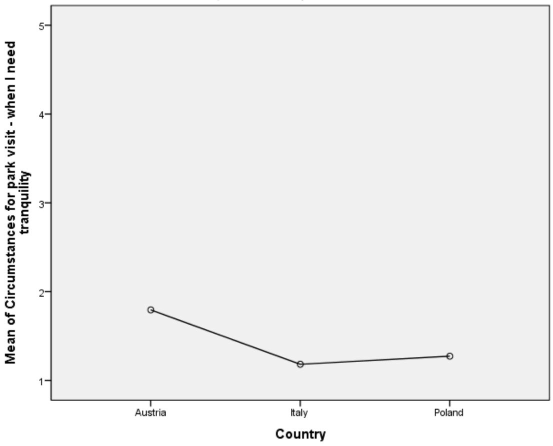

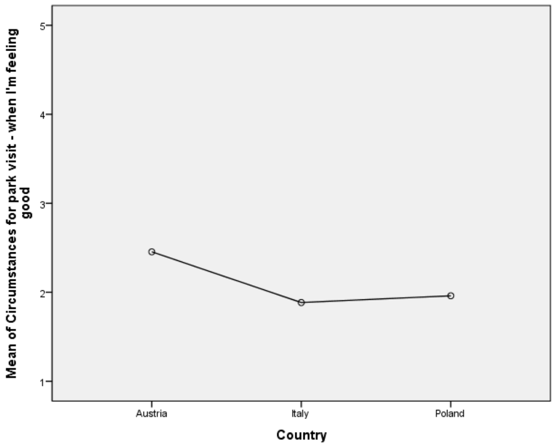

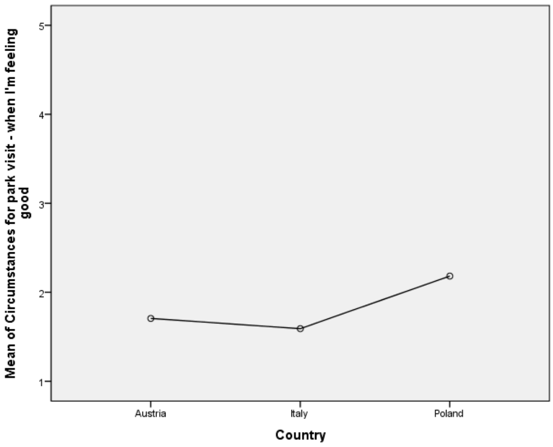

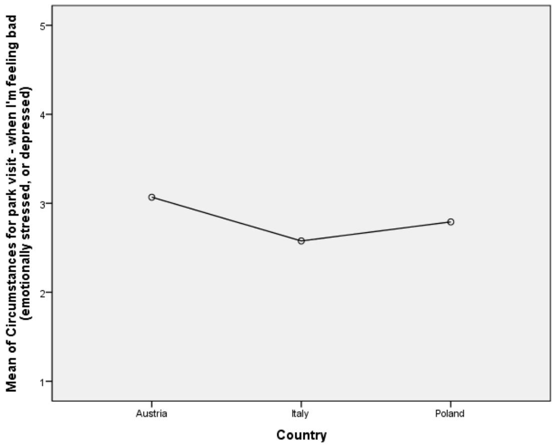

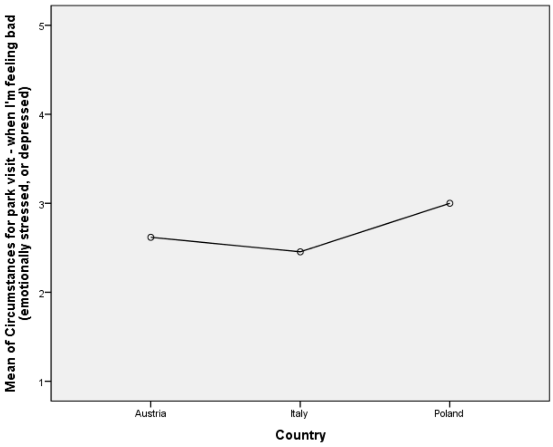

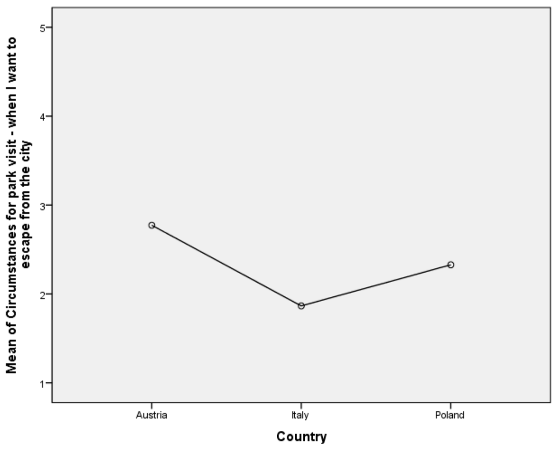

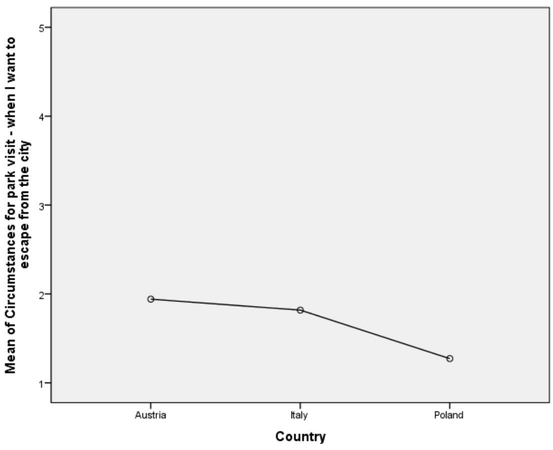

10. Relationships between the circumstances for PA visit and country (for visitor age category < 24 years)

ANOVA

		Sum of Squares	df	Mean Square	F
Circumstances for park visit - when I need tranquility	Between Groups	53,326	2	26,663	20,226
	Within Groups	413,929	314	1,318	
	Total	467,256	316		
Circumstances for park visit - when I'm feeling good	Between Groups	16,851	2	8,426	8,650
	Within Groups	305,849	314	,974	
	Total	322,700	316		
Circumstances for park visit - when I'm feeling bad (emotionally stressed, or depressed)	Between Groups	8,590	2	4,295	2,700
	Within Groups	499,549	314	1,591	
	Total	508,139	316		
Circumstances for park visit - when I want to escape from the city	Between Groups	27,821	2	13,911	8,692
	Within Groups	502,507	314	1,600	
	Total	530,328	316		

ANOVA

		Sig.
Circumstances for park visit - when I need tranquility	Between Groups	,000
	Within Groups	
	Total	
Circumstances for park visit - when I'm feeling good	Between Groups	,000
	Within Groups	
	Total	
Circumstances for park visit - when I'm feeling bad (emotionally stressed, or depressed)	Between Groups	,069
	Within Groups	
	Total	

		Sig.
Circumstances for park visit - when I want to escape from the city	Between Groups	,000
	Within Groups	
	Total	

11. **Relationships between the circumstances for PA visit and country (for visitor age category > 65 years)**

Oneway

ANOVA

		Sum of Squares	df	Mean Square	F
Circumstances for park visit - when I need tranquility	Between Groups	5,733	2	2,866	8,730
	Within Groups	21,013	64	,328	
	Total	26,746	66		
Circumstances for park visit - when I'm feeling good	Between Groups	2,673	2	1,337	2,138
	Within Groups	40,013	64	,625	
	Total	42,687	66		
Circumstances for park visit - when I'm feeling bad (emotionally stressed, or depressed)	Between Groups	2,188	2	1,094	,765
	Within Groups	91,484	64	1,429	
	Total	93,672	66		
Circumstances for park visit - when I want to escape from the city	Between Groups	3,738	2	1,869	2,527
	Within Groups	47,337	64	,740	
	Total	51,075	66		

ANOVA

		Sig.
Circumstances for park visit - when I need tranquility	Between Groups	,000
	Within Groups	
	Total	
Circumstances for park visit - when I'm feeling good	Between Groups	,126
	Within Groups	
	Total	
Circumstances for park visit - when I'm feeling bad (emotionally stressed, or depressed)	Between Groups	,469
	Within Groups	
	Total	
Circumstances for park visit - when I want to escape from the city	Between Groups	,088
	Within Groups	
	Total	

12. **Relationships between the symptoms that can be reduced by contact with nature and country (for visitor age category < 24 years)**

Oneway

ANOVA

		Sum of Squares	df	Mean Square	F
Symptoms that can be reduced by contact with nature - Iritability	Between Groups	3,415	2	1,707	2,527
	Within Groups	211,522	313	,676	
	Total	214,937	315		
Symptoms that can be reduced by contact with nature - Lack of ability to concentrate	Between Groups	,345	2	,173	,206
	Within Groups	262,427	313	,838	
	Total	262,772	315		
Symptoms that can be reduced by contact with nature - Insomnia	Between Groups	,213	2	,106	,124
	Within Groups	268,332	313	,857	
	Total	268,544	315		
Symptoms that can be reduced by contact with nature - Muscolar tension	Between Groups	5,132	2	2,566	2,165
	Within Groups	370,944	313	1,185	
	Total	376,076	315		
Symptoms that can be reduced by contact with nature - Pain	Between Groups	9,132	2	4,566	4,533
	Within Groups	315,308	313	1,007	
	Total	324,440	315		
Symptoms that can be reduced by contact with nature - General anxiety	Between Groups	4,177	2	2,088	2,374
	Within Groups	274,439	312	,880	
	Total	278,616	314		
Symptoms that can be reduced by contact with nature - Depression	Between Groups	,334	2	,167	,181
	Within Groups	288,856	313	,923	
	Total	289,190	315		
Symptoms that can be reduced by contact with nature - Short term memory	Between Groups	,562	2	,281	,230
	Within Groups	383,905	314	1,223	
	Total	384,467	316		

ANOVA

		Sig.
Symptoms that can be reduced by contact with nature - Iritability	Between Groups	,082
	Within Groups	
	Total	
Symptoms that can be reduced by contact with nature - Lack of ability to concentrate	Between Groups	,814
	Within Groups	
	Total	
Symptoms that can be reduced by contact with	Between Groups	,883

nature - Insomnia	Within Groups	
	Total	
Symptoms that can be reduced by contact with nature - Muscolar tension	Between Groups	,116
	Within Groups	
	Total	
Symptoms that can be reduced by contact with nature - Pain	Between Groups	,011
	Within Groups	
	Total	
Symptoms that can be reduced by contact with nature - General anxiety	Between Groups	,095
	Within Groups	
	Total	
Symptoms that can be reduced by contact with nature - Depression	Between Groups	,835
	Within Groups	
	Total	
Symptoms that can be reduced by contact with nature - Short term memory	Between Groups	,795
	Within Groups	
	Total	

13. **Relationships between the symptoms that can be reduced by contact with nature and country (for visitor age category > 65 years)**

Oneway

ANOVA

		Sum of Squares	df	Mean Square	F
Symptoms that can be reduced by contact with nature - Iritability	Between Groups	1,396	2	,698	1,424
	Within Groups	31,380	64	,490	
	Total	32,776	66		
Symptoms that can be reduced by contact with nature - Lack of ability to concentrate	Between Groups	1,904	2	,952	2,211
	Within Groups	27,559	64	,431	
	Total	29,463	66		
Symptoms that can be reduced by contact with nature - Insomnia	Between Groups	2,468	2	1,234	2,760
	Within Groups	28,607	64	,447	
	Total	31,075	66		
Symptoms that can be reduced by contact with nature - Muscolar tension	Between Groups	8,156	2	4,078	7,572
	Within Groups	34,471	64	,539	
	Total	42,627	66		
Symptoms that can be reduced by contact with nature - Pain	Between Groups	4,819	2	2,409	2,604
	Within Groups	59,211	64	,925	
	Total	64,030	66		
Symptoms that can be reduced by contact with nature - General anxiety	Between Groups	,758	2	,379	1,081
	Within Groups	22,436	64	,351	
	Total	23,194	66		
Symptoms that can be reduced by contact with nature - Depression	Between Groups	1,560	2	,780	1,360
	Within Groups	36,709	64	,574	
	Total	38,269	66		
Symptoms that can be reduced by contact with nature - Short term memory	Between Groups	4,860	2	2,430	2,600
	Within Groups	59,826	64	,935	
	Total	64,687	66		

ANOVA

		Sig.
Symptoms that can be reduced by contact with nature - Iritability	Between Groups	,248
	Within Groups	
	Total	
Symptoms that can be reduced by contact with nature - Lack of ability to concentrate	Between Groups	,118
	Within Groups	
	Total	
Symptoms that can be reduced by contact with	Between Groups	,071

nature - Insomnia	Within Groups	
	Total	
Symptoms that can be reduced by contact with nature - Muscolar tension	Between Groups	,001
	Within Groups	
	Total	
Symptoms that can be reduced by contact with nature - Pain	Between Groups	,082
	Within Groups	
	Total	
Symptoms that can be reduced by contact with nature - General anxiety	Between Groups	,345
	Within Groups	
	Total	
Symptoms that can be reduced by contact with nature - Depression	Between Groups	,264
	Within Groups	
	Total	
Symptoms that can be reduced by contact with nature - Short term memory	Between Groups	,082
	Within Groups	
	Total	

**Means Plots** Age <24	Age >65

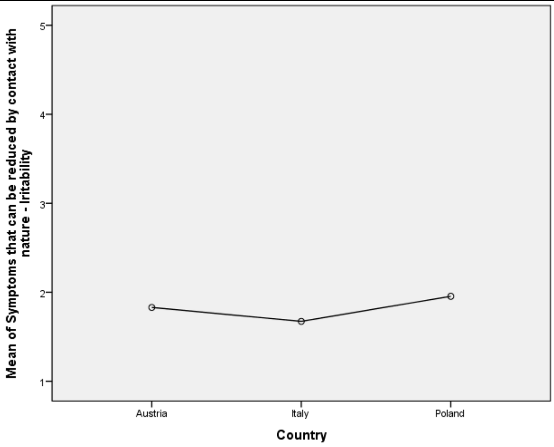

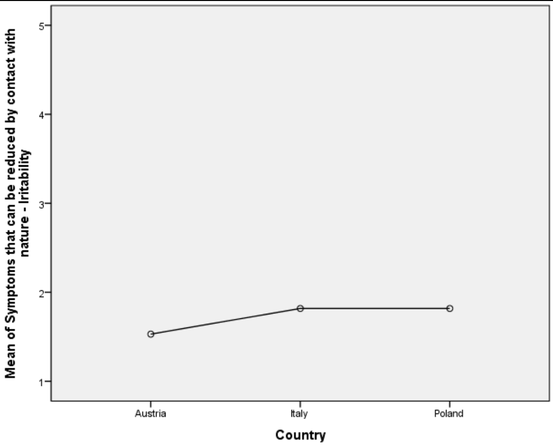

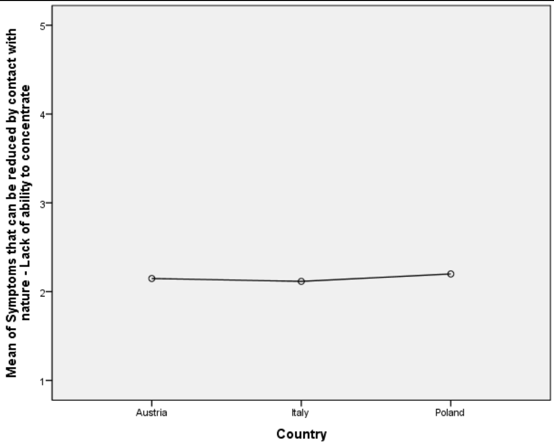

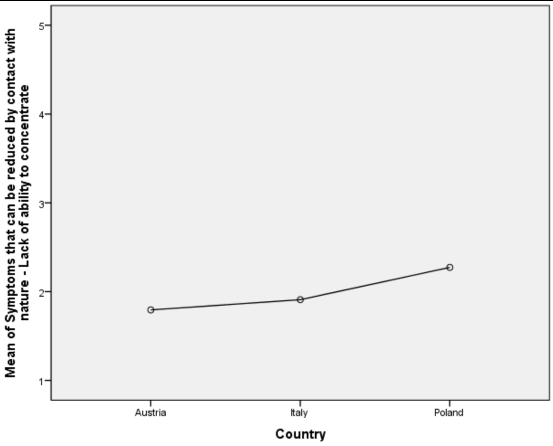

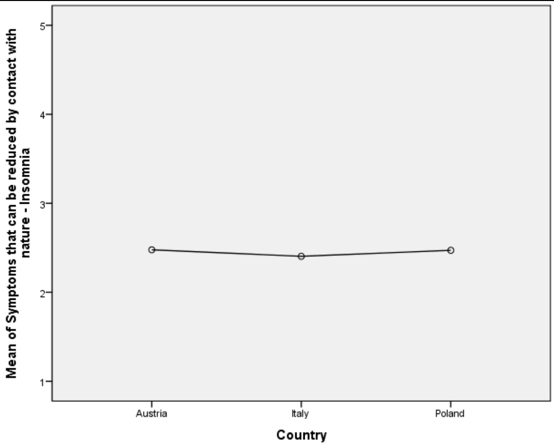

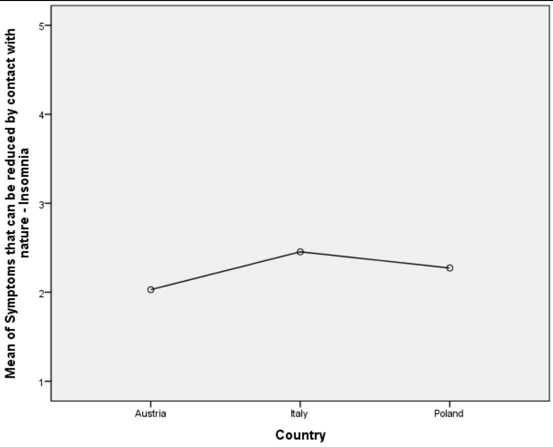

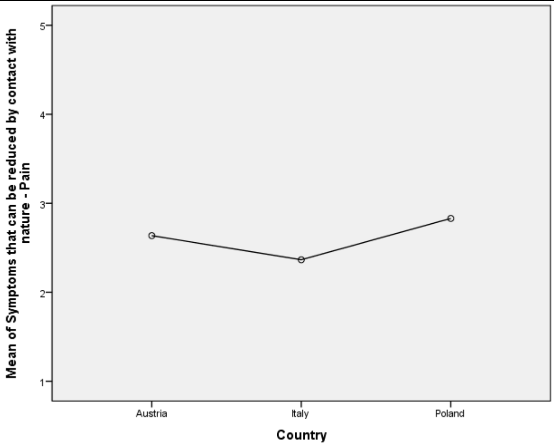

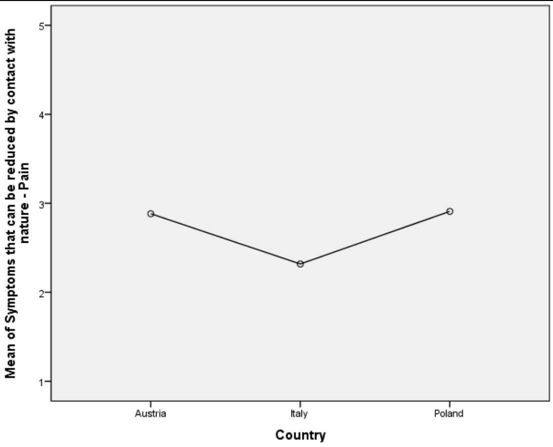

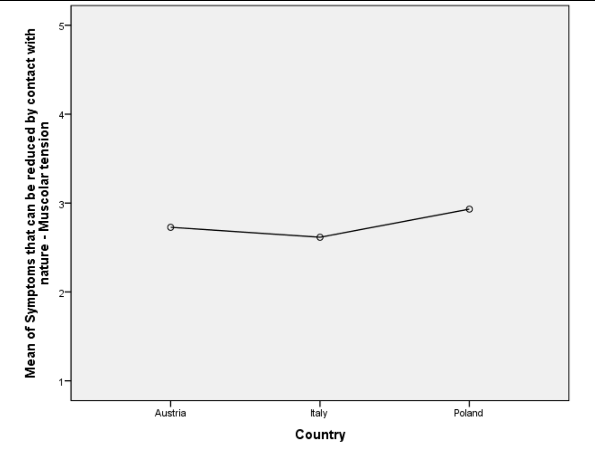

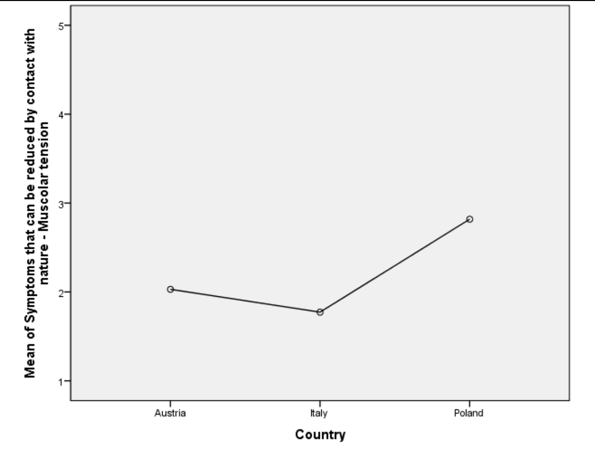

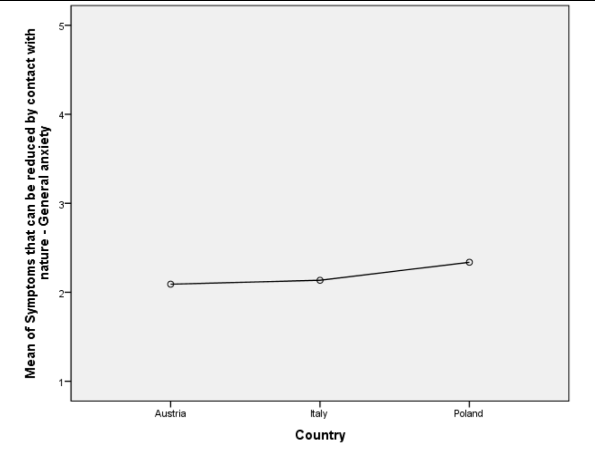

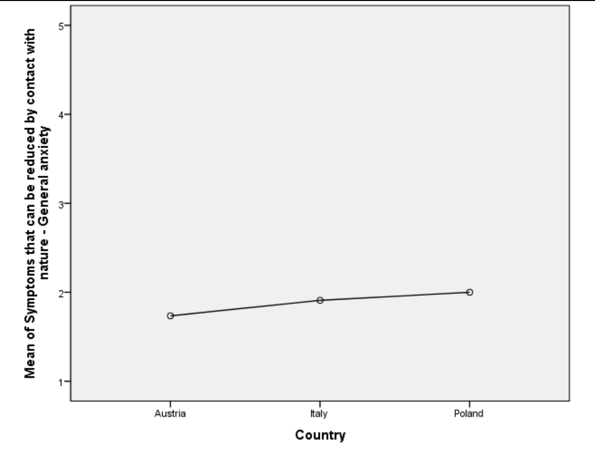

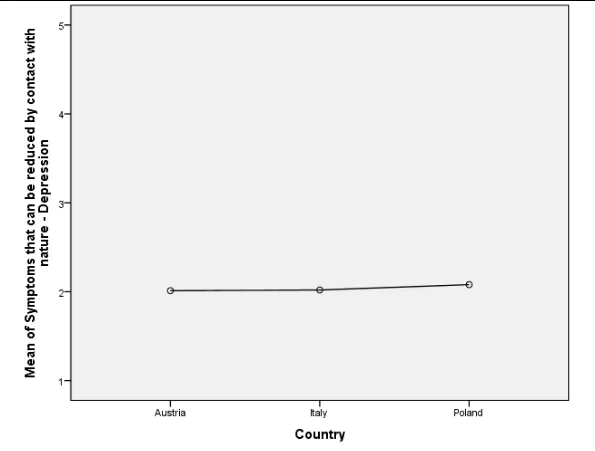

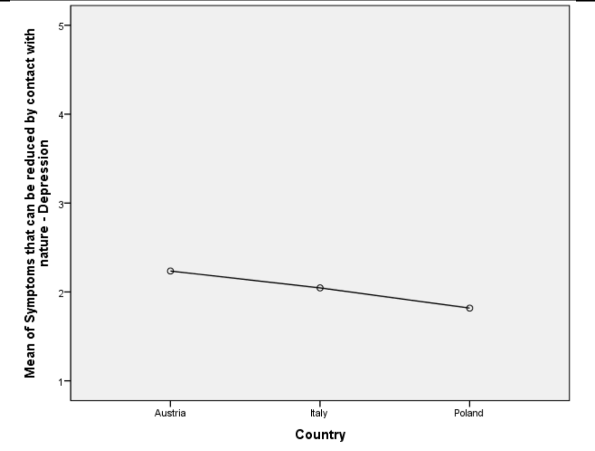

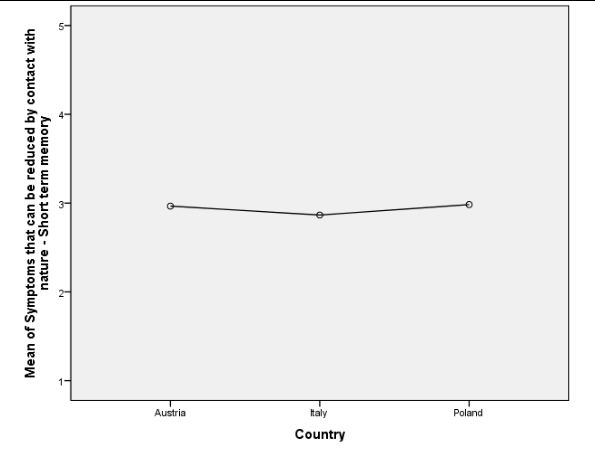

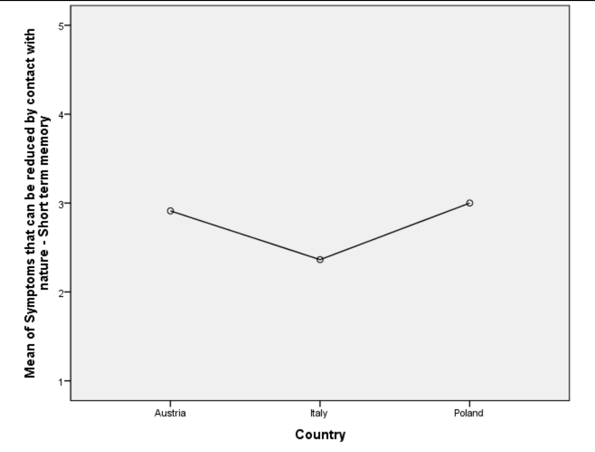

Post Hoc Tests

14. **Relationships between the circumstances for PA visit and country (whole sample; results of post-hoc tests)**

Multiple Comparisons

Dependent Variable		(I) Country	(J) Country	Mean Difference (I-J)	Std. Error	Sig.	95% Confidence Interval Lower Bound	95% Confidence Interval Upper Bound
Circumstances for park visit - when I need tranquility	LSD	Austria	Italy	,537*	,067	,000	,40	,67
			Poland	,420*	,067	,000	,29	,55
		Italy	Austria	-,537*	,067	,000	-,67	-,40
			Poland	-,117	,068	,085	-,25	,02
		Poland	Austria	-,420*	,067	,000	-,55	-,29
			Italy	,117	,068	,085	-,02	,25
	Bonferroni	Austria	Italy	,537*	,067	,000	,38	,70
			Poland	,420*	,067	,000	,26	,58
		Italy	Austria	-,537*	,067	,000	-,70	-,38
			Poland	-,117	,068	,255	-,28	,05
		Poland	Austria	-,420*	,067	,000	-,58	-,26
			Italy	,117	,068	,255	-,05	,28
Circumstances for park visit - when I'm feeling good	LSD	Austria	Italy	,209*	,058	,000	,09	,32
			Poland	,158*	,058	,007	,04	,27
		Italy	Austria	-,209*	,058	,000	-,32	-,09
			Poland	-,051	,059	,388	-,17	,06
		Poland	Austria	-,158*	,058	,007	-,27	-,04
			Italy	,051	,059	,388	-,06	,17
	Bonferroni	Austria	Italy	,209*	,058	,001	,07	,35
			Poland	,158*	,058	,020	,02	,30
		Italy	Austria	-,209*	,058	,001	-,35	-,07
			Poland	-,051	,059	1,000	-,19	,09
		Poland	Austria	-,158*	,058	,020	-,30	-,02
			Italy	,051	,059	1,000	-,09	,19
Circumstances for park visit - when I'm feeling bad (emotionally stressed, or depressed)	LSD	Austria	Italy	,232*	,080	,004	,07	,39
			Poland	,208*	,080	,009	,05	,37
		Italy	Austria	-,232*	,080	,004	-,39	-,07
			Poland	-,024	,081	,769	-,18	,13
		Poland	Austria	-,208*	,080	,009	-,37	-,05
			Italy	,024	,081	,769	-,13	,18
	Bonferroni	Austria	Italy	,232*	,080	,012	,04	,42
			Poland	,208*	,080	,028	,02	,40
		Italy	Austria	-,232*	,080	,012	-,42	-,04

			Poland	-,024	,081	1,000	-,22	,17
		Poland	Austria	-,208*	,080	,028	-,40	-,02
			Italy	,024	,081	1,000	-,17	,22
Circumstances for park visit - when I want to escape from the city	LSD	Austria	Italy	,318*	,080	,000	,16	,48
			Poland	-,230*	,080	,004	-,39	-,07
		Italy	Austria	-,318*	,080	,000	-,48	-,16
			Poland	-,548*	,081	,000	-,71	-,39
		Poland	Austria	,230*	,080	,004	,07	,39
			Italy	,548*	,081	,000	,39	,71
	Bonferroni	Austria	Italy	,318*	,080	,000	,12	,51
			Poland	-,230*	,080	,013	-,42	-,04
		Italy	Austria	-,318*	,080	,000	-,51	-,12
			Poland	-,548*	,081	,000	-,74	-,35
		Poland	Austria	,230*	,080	,013	,04	,42
			Italy	,548*	,081	,000	,35	,74

*. The mean difference is significant at the 0.05 level.
